# Real-World Validation of PinPoint Blood Tests in the NHS: Multivariable Machine Learning to Predict Cancer Risk in Primary Care Urgent Referrals

**DOI:** 10.1016/j.mcpdig.2026.100382

**Published:** 2026-06-10

**Authors:** Matt Neal, Mark Dean, Sean Duffy, Roisean E. Ferguson, Peter Horan, Colin Johnston, Katherine L. Lloyd, Deehan Mair, Richard Neal, Jeanette Riley, Helen Ryan, Nigel Sansom, Richard S. Savage, Nisha Sharma, Jim Skinner, Giles Tully, Michelle Wilson, Will Worboys

**Affiliations:** a PinPoint Data Science Ltd, Nexus, Discovery Way, West Yorkshire, United Kingdom; b Leeds Institute of Medical Research, University of Leeds, West Yorkshire, United Kingdom; c Department of Health and Community Sciences, University of Exeter, St Luke's Campus, Devon, United Kingdom; d Mid Yorkshire Teaching NHS Trust, West Yorkshire, United Kingdom; e The West Yorkshire and Harrogate Cancer Alliance, White Rose House, United Kingdom; f The Leeds Teaching Hospitals NHS Trust, St James’s University Hospital, West Yorkshire, United Kingdom; g Calderdale and Huddersfield NHS Foundation Trust, West Yorkshire, United Kingdom

## Abstract

**Objective:**

To validate the United Kingdom Conformity Assessed-marked PinPoint blood tests, which use machine learning models and routinely available blood analytes to estimate cancer risk in adults referred on urgent suspected cancer pathways in National Health Service (NHS) England.

**Patients and Methods:**

This work comprises a large-scale, prospective, observational, real-world NHS service evaluation of 9 blood tests, carried out from December 21, 2020 to July 31, 2025. Total of 16,481 patients with urgent suspected cancer referrals were enrolled across 5 secondary care Trusts and 170 General Practitioner surgeries. Real-world performance of the tests was evaluated using a range of diagnostic accuracy statistics.

**Results:**

Five tests have performance indicating potential clinical utility. Receiver operating characteristic area-under-curve scores (95% CI) for these were: Upper gastrointestinal=0.86 (0.81-0.90), Gynecological=0.81 (0.77-0.85), Lung=0.79 (0.74-0.84), Head & Neck=0.73 (0.68-0.78), and Lower gastrointestinal=0.72 (0.67-0.78), Prioritization of the 10% of highest-risk patients would reduce the number needed to investigate to detect one cancer by a factor of 2.6-6.1.

**Conclusion:**

This work shows the potential of these tests to improve urgent suspected cancer referral pathways. High-risk patients could be diagnosed more rapidly, leading to potential earlier-stage diagnosis and a better diagnostic experience. Low-risk patients could avoid unnecessary invasive medical testing for cancer. The software can be deployed rapidly across the NHS, without the need for additional hardware.

Urgent referrals from primary care are a major route for cancer diagnosis in the National Health service (NHS), with more than 3 million patients referred annually in England alone. The annual referral rate has increased 10% year-on-year for the last 15 years,^1^ and the urgent suspected cancer (USC) pathways have an average conversion rate of only 6%, meaning that improved methods of triage are urgently needed.

This ongoing increase in referral rates places huge strain on the system.^2^ The USC pathways have time targets, which are put at risk by rising workload, limited physical capacity, and constrained budgets. Improved methods for triage would allow patients with a higher likelihood of cancer to be prioritized for investigation, resulting in an enriched referral population with higher cancer prevalence. It would also allow the identification and rapid reassurance of patients at much lower risk, who could then be managed in primary care and avoid unnecessary invasive medical testing for suspected cancer.

The PinPoint Tests are a set of United Kingdom Conformity Assessed-marked multi-cancer early detection blood tests for predicting the cancer risk of symptomatic patients.^3^ This paper describes the result of an NHS service evaluation of the 9 PinPoint Tests in West Yorkshire and Harrogate from December 2020 to July 2025. The goals of the work were (i) to validate the diagnostic accuracy of the tests in a prospective, real-world setting, and (ii) to report the feasibility of deploying the technology in a health care setting.

## Patients And Methods

### Methodological Design and Source of Data

This was a prospective, observational, multicenter service evaluation assessing the diagnostic accuracy of the PinPoint Tests. Five NHS trusts participated, joining enrollment on different dates: Mid Yorkshire Teaching NHS Trust (MYTT), Leeds Teaching Hospitals NHS Trust, Calderdale and Huddersfield NHS Foundation Trust, Bradford Teaching Hospitals NHS Foundation Trust and Harrogate and District NHS Foundation Trust. Patients were enrolled consecutively in both primary and secondary care settings as part of an USC referral visit. Staff were instructed to try to enroll every eligible patient.

Patient-level consent was obtained as part of enrollment. The work was carried out under a service evaluation with formal approval from West Yorkshire Research and Development Office (Ref no: 001_02_07_2020_0000 (Service evaluation)), and with specific approval for data sharing from the Trust Caldicott Guardian of participating Acute Trusts.

Patient enrollment began on December 21, 2020. Enrollment end dates varied by pathway, as listed in the Supplemental Appendix (available online at https://www.mcpdigitalhealth.org/) and all pathways completed by July 31, 2025.

The appropriate PinPoint Test was ordered through standard primary or secondary care processes at enrollment. Blood samples were obtained by phlebotomists or appropriately trained nurses and processed at the MYTT NHS pathology laboratory using equipment in routine clinical use. Results were transmitted automatically to the PinPoint algorithm, hosted by The Health Informatics Service on NHS IT infrastructure. Patient outcomes were extracted from electronic health records by Trust data analysts.

Clinicians were blinded to the results; the patient’s onward journey was unaffected.

### Participants

The patient inclusion criteria were: aged 18 years or older; referred to one of 9 USC pathways (breast, gynecologic, hematologic, head and neck, lung, lower gastrointestinal, skin, upper gastrointestinal, and urological). Referral criteria for the USC pathways are described in NICE guideline NG12.^4^ No additional exclusion criteria were applied.

### Outcomes

Patient diagnoses were determined by the outcome of their USC referral, with deidentified results provided by NHS data analysts. Completed referrals were those determined to have been concluded by clinicians as part of standard care. Most patients referred to a USC pathway will have completed the diagnostic process within 3 months. Patients were included in the final analyses if their referral was completed within 3 months of the end of enrollment of patients in the relevant pathway. If a cancer diagnosis was not recorded the patient was allocated to the non-cancer class. The ICD-10 codes that were used to define the cancer class were previously described.^3^

### Predictors

The predictors are age, sex, and a panel of standard blood analytes, including full blood count, liver function tests, urea and electrolytes, bone profile, inflammatory markers, and a set of tumor markers. A full list of predictors is included in the Supplemental Appendix.

Patient ethnicity was collected to evaluate differences in test performance for different ethnic groups, coded using NHS ethnicity definitions.^5^

### Sample Size

The sample size calculation method is given in the Supplemental Appendix.

### Bias

The service evaluation commenced during the COVID-19 pandemic, which affected referral numbers and demographic characteristics in ways that could not be adjusted for.^6^^,^^7^

**Figure 1:**
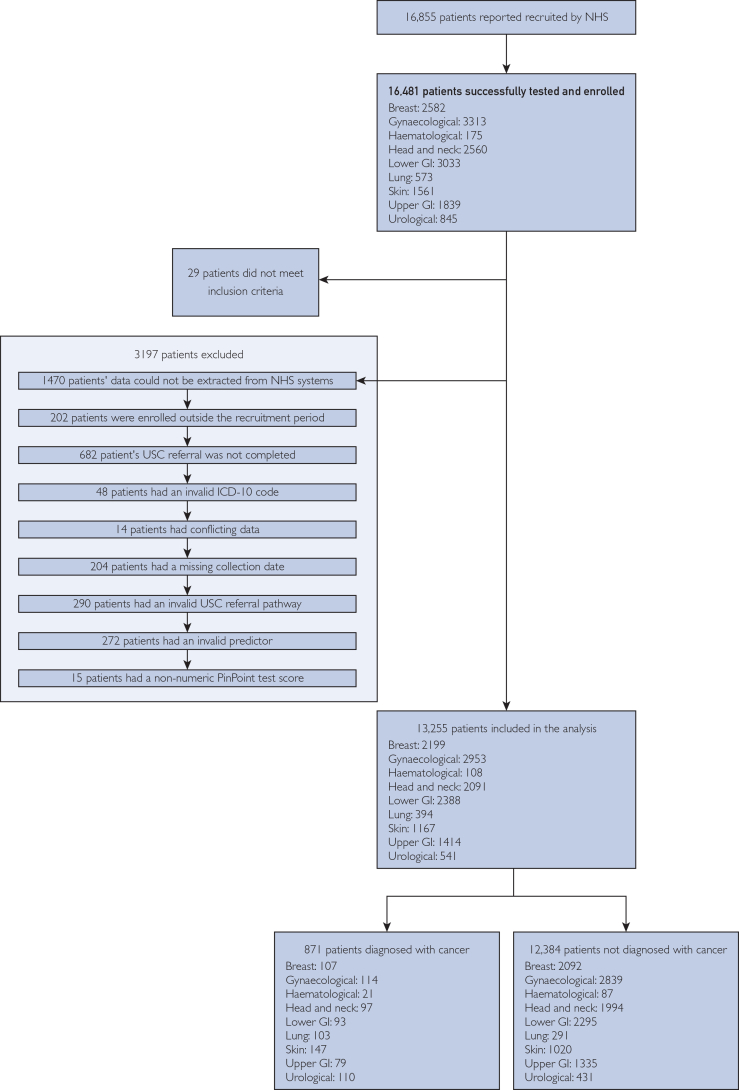
Flow chart of patient inclusion and exclusion.

About 3,197 (19.4%) patients were excluded from the analysis for various reasons. Figure 1 shows a breakdown of these reasons. A sensitivity analysis was carried out to quantify the reasonable worst-case impact resulting from these exclusions, the details of which are given in the Supplemental Appendix.

### Management of Missing Data

Patients with missing data in the predictors (analyte values, age, and sex) were excluded from the analysis. If a referral was incomplete, the patient was considered lost to follow-up and excluded. If all analyte values and outcomes were present but no PinPoint Test result was available, the algorithm was used to produce a test result. If predictors, outcome, and PinPoint Test results were available but ethnicity was missing, the patient was included in the primary analysis but excluded from demographic characteristic analyses regarding ethnicity.

### Statistical Analysis Methods

#### Model Development

Model development for v1.1 was previously described.^3^ As per the protocol, an updated version (v1.2) of the algorithm was developed in parallel to the service evaluation. The v1.2 tests are improved tests, using the same retrospective data as previously presented,^3^ but using the entire data set to train the models. Three additional elements were also added, (1) use of hot-deck imputation to address missing training data values, (2) re-introducing the relevant sparse features by ensembling the retrained multivariate model with monotonic univariate models, and (3) calibration correction for differences between training data prevalence and expected prevalence in the USC pathways. No other differences were introduced.

#### Analysis Methods

Analysis was performed using Python (version 3.11.4) and R (version 4.3.1). The performance of the 2 sets of models described above was investigated. The results for both model versions used the same input measurements for each patient.

Performance metrics are reported as point estimates and 95% CIs. The receiver operating characteristic area-under-curve (AUC) CIs were calculated using DeLong’s method.^8^ Wilson CIs were used for binomial proportions, such as sensitivity, specificity, negative predictive value, and positive predictive value. Calibration was assessed using calibration curves and observed-to-expected (O/E) ratios.

Patient age, sex, and ethnicity were collected at patient enrollment. Subgroup analyses were performed to investigate the impact of these variables. Inclusion was restricted to those for whom the relevant information was provided.

Temporal analysis was performed to assess drift in prevalence and performance across the service evaluation. Details can be found in the Supplemental Appendix, available online at https://www.mcpdigitalhealth.org). All analyses were stratified by USC pathway.

Diagnostic accuracy analysis was conducted for 3 illustrative use cases for each pathway.

1. 20% Rule-Out: threshold set so that 20% of disease-negative patients with the lowest test scores are identified as test-negative.
2. 90% Rule-In: threshold set so that 90% of cancer cases are identified as test-positive.
3. 10% Prioritization: threshold set so that 10% of patients with the highest test scores are identified as test-positive.

### Ethics Statement

The design of the service evaluation was formally approved by the West Yorkshire Research and Development Team in the West Yorkshire Integrated Care Board. All patients gave recorded verbal consent, as per the requirement for this NHS service evaluation.

## Results

This work produced results in nine USC referral pathways, evaluating test performance when deployed to a real-world health care setting. The patients had all been referred to a USC pathway and had blood taken for this work at the start of that referral journey, with the goal of making these results as close to routine clinical practice as possible.

Five tests have performance that show potential clinical utility, with ROC curve AUCs as high as 0.86. For the prioritization use case, the number needed to investigate to detect one cancer reduced by a factor of 2.6-6.1. Four of these five tests achieved an negative predictive value >0.99 when used to rule-out 20% of the lowest-risk patients.

### Participants & Demographic Characteristics

Figure 1 shows the flow of patients, with the number of patients at each stage. Of the 16,481 patients enrolled, 29 (0.2%) were ineligible per the protocol’s age criterion, and a further 3,197 (19.4%) were excluded for the reasons shown in Figure 1, leaving 13,255 (80.4%) in the primary analysis. Of the remaining patients, 871 (6.6%) were diagnosed with cancer and 12,384 (93.4%) with non-cancer diseases or no disease.

Table 1 contains demographic information and clinical characteristics for the included patients, split by referral pathway. Tables showing the diagnoses observed in the included patients are given in the Supplemental Appendix. These are in keeping with expected numbers.

**Table 1:** Patient Demographic Characteristics and Clinical Characteristics, Split by USC Pathway

	Breast	Gynecological	Hematological	Head and Neck	Lower GI	Lung	Skin	Upper GI	Urological
Total Patients, n	2199	2953	108	2091	2388	394	1167	1414	541
Age, y									
Median	48	56	66	61	68	69	67	67	68
IQR	38-60	51-64	54-75	48-72	58-76	59-76	53-75	56-75	61-75
Missing	0	0	0	0	0	0	0	0	0
Sex, n (%)									
Male	104 (4.7%)	1 (0.0%)	59 (54.6%)	831 (39.7%)	1105 (46.3%)	215 (54.6%)	553 (47.4%)	552 (39.0%)	467 (86.3%)
Female	2095 (95.3%)	2952 (100.0%)	49 (45.4%)	1260 (60.3%)	1283 (53.7%)	179 (45.4%)	614 (52.6%)	862 (61.0%)	74 (13.7%)
Missing	0 (0.0%)	0 (0.0%)	0 (0.0%)	0 (0.0%)	0 (0.0%)	0 (0.0%)	0 (0.0%)	0 (0.0%)	0 (0.0%)
Ethnicity, n (%)									
White	707 (32.2%)	1039 (35.2%)	53 (49.1%)	1111 (53.1%)	736 (30.8%)	194 (49.2%)	608 (52.1%)	633 (44.8%)	486 (89.8%)
Asian/Asian British	26 (1.2%)	52 (1.8%)	1 (0.9%)	65 (3.1%)	33 (1.4%)	4 (1.0%)	4 (0.3%)	46 (3.3%)	26 (4.8%)
Black/African/Caribbean/Black British	13 (0.6%)	20 (0.7%)	0 (0.0%)	12 (0.6%)	10 (0.4%)	7 (1.8%)	3 (0.3%)	5 (0.4%)	8 (1.5%)
Other ethnic group	12 (0.5%)	20 (0.7%)	2 (1.9%)	21 (1.0%)	9 (0.4%)	0 (0.0%)	11 (0.9%)	8 (0.6%)	5 (0.9%)
Missing	1441 (65.5%)	1822 (61.7%)	52 (48.1%)	882 (42.2%)	1600 (67.0%)	189 (48.0%)	541 (46.4%)	722 (51.1%)	16 (3.0%)
Outcome, n (%)									
Cancer	107 (4.9%)	114 (3.9%)	21 (19.4%)	97 (4.6%)	93 (3.9%)	103 (26.1%)	147 (12.6%)	79 (5.6%)	110 (20.3%)
Non-cancer	2092 (95.1%)	2839 (96.1%)	87 (80.6%)	1994 (95.4%)	2295 (96.1%)	291 (73.9%)	1020 (87.4%)	1335 (94.4%)	431 (79.7%)
Missing	0 (0.0%)	0 (0.0%)	0 (0.0%)	0 (0.0%)	0 (0.0%)	0 (0.0%)	0 (0.0%)	0 (0.0%)	0 (0.0%)

Abbreviations: GI, gastrointestinal; USC, urgent suspected cancer.

### Performance by Pathway

Table 2, Table 3, Table 4 show the performance metrics for each v1.2 PinPoint Test for 3 use cases. Cross-tabulations of PinPoint Test results versus cancer/non-cancer diagnosis for each pathway and use case can be found in the Supplemental Appendix. The key findings are that 5 tests (upper GI, gynecological, lung, head and neck, and lower GI) have performance that show potential clinical utility, with ROC AUCs ranging from 0.72 to 0.86. If used for prioritization of the top 10% of highest-risk patients, these tests would reduce the number needed to investigate to detect one cancer by a factor of 2.6 to 6.1.

**Table 2:** Performance Characteristics for PinPoint Test Version 1.2. Aim: 20% Rule-Out

Pathway	AUC (95% CI)	NPV (95% CI)	Sensitivity (95% CI)	Specificity (95% CI)	PPV (95% CI)	Threshold
Breast	0.73 (0.68-0.78)	0.99 (0.97-0.99)	0.94 (0.88-0.98)	0.20 (0.18-0.22)	0.06 (0.05-0.07)	0.05
Gynecologic	0.81 (0.77-0.85)	1.00 (0.99-1.00)	0.99 (0.95-1.00)	0.20 (0.19-0.22)	0.05 (0.04-0.06)	0.03
Hematologic	0.67 (0.55-0.78)	1.00 (0.77-1.00)	1.00 (0.81-1.00)	0.20 (0.12-0.30)	0.23 (0.15-0.33)	0.10
Head and neck	0.73 (0.68-0.78)	0.99 (0.97-1.00)	0.96 (0.89-0.99)	0.20 (0.18-0.22)	0.06 (0.04-0.07)	0.02
Lower GI	0.72 (0.67-0.78)	0.99 (0.97-1.00)	0.95 (0.87-0.98)	0.20 (0.18-0.22)	0.05 (0.04-0.06)	0.03
Lung	0.79 (0.74-0.84)	0.95 (0.85-0.99)	0.97 (0.91-0.99)	0.20 (0.16-0.25)	0.30 (0.25-0.35)	0.13
Skin	0.62 (0.57-0.66)	0.94 (0.89-0.96)	0.90 (0.84-0.95)	0.20 (0.18-0.23)	0.14 (0.12-0.16)	0.09
Upper GI	0.86 (0.81-0.90)	0.99 (0.97-1.00)	0.97 (0.90-1.00)	0.20 (0.18-0.22)	0.07 (0.05-0.08)	0.03
Urological	0.73 (0.68-0.79)	0.96 (0.88-0.99)	0.96 (0.90-0.99)	0.20 (0.16-0.24)	0.24 (0.20-0.28)	0.19

Abbreviations: AUC, area-under-curve; GI, gastro intestinal; NPV, negative predictive value; PPV, positive predictive value.

**Table 3:** Performance Characteristics for PinPoint Test Version 1.2. Aim: 90% Rule-in

Pathway	AUC (95% CI)	NPV (95% CI)	Sensitivity (95% CI)	Specificity (95% CI)	PPV (95% CI)	Threshold
Breast	0.73 (0.68-0.78)	0.98 (0.97-0.99)	0.90 (0.82-0.95)	0.32 (0.30-0.34)	0.06 (0.05-0.08)	0.05
Gynecologic	0.81 (0.77-0.85)	0.99 (0.98-1.00)	0.89 (0.82-0.94)	0.48 (0.46-0.50)	0.06 (0.05-0.08)	0.04
Hematologic	0.67 (0.55-0.78)	0.93 (0.79-0.98)	0.86 (0.63-0.96)	0.43 (0.32-0.54)	0.26 (0.17-0.39)	0.15
Head and neck	0.73 (0.68-0.78)	0.99 (0.98-0.99)	0.90 (0.81-0.95)	0.41 (0.38-0.43)	0.07 (0.06-0.08)	0.02
Lower GI	0.72 (0.67-0.78)	0.99 (0.98-0.99)	0.89 (0.81-0.94)	0.33 (0.31-0.35)	0.05 (0.04-0.06)	0.03
Lung	0.79 (0.74-0.84)	0.91 (0.83-0.95)	0.89 (0.81-0.94)	0.36 (0.31-0.42)	0.33 (0.28-0.39)	0.15
Skin	0.62 (0.57-0.66)	0.93 (0.89-0.96)	0.90 (0.83-0.94)	0.21 (0.18-0.23)	0.14 (0.12-0.16)	0.09
Upper GI	0.86 (0.81-0.90)	0.99 (0.98-1.00)	0.90 (0.81-0.95)	0.56 (0.53-0.58)	0.11 (0.09-0.13)	0.04
Urological	0.73 (0.68-0.79)	0.93 (0.87-0.96)	0.89 (0.81-0.94)	0.35 (0.30-0.39)	0.26 (0.22-0.31)	0.21

Abbreviations: AUC, area-under-curve; GI, gastro intestinal; NPV, negative predictive value; PPV, positive predictive value.

**Table 4:** Performance Characteristics for PinPoint Test Version 1.2. Aim: 10% Prioritization

Pathway	AUC (95% CI)	NPV (95% CI)	Sensitivity (95% CI)	Specificity (95% CI)	PPV (95% CI)	Reduction in number of tests to detect one cancer	Threshold
Breast	0.73 (0.68-0.78)	0.96 (0.95-0.97)	0.33 (0.24-0.43)	0.91 (0.90-0.92)	0.16 (0.11-0.22)	3.3×	0.08
Gynecologic	0.81 (0.77-0.85)	0.97 (0.97-0.98)	0.41 (0.32-0.51)	0.91 (0.90-0.92)	0.16 (0.12-0.21)	4.1×	0.06
Hematologic	0.67 (0.55-0.78)	0.80 (0.71-0.88)	0.10 (0.02-0.32)	0.90 (0.81-0.95)	0.18 (0.03-0.52)	0.94×	0.45
Head and Neck	0.73 (0.68-0.78)	0.97 (0.96-0.97)	0.34 (0.25-0.44)	0.91 (0.90-0.92)	0.16 (0.11-0.22)	3.5×	0.05
Lower GI	0.72 (0.67-0.78)	0.97 (0.96-0.98)	0.34 (0.25-0.45)	0.91 (0.90-0.92)	0.13 (0.09-0.19)	3.3×	0.06
Lung	0.79 (0.74-0.84)	0.79 (0.74-0.83)	0.26 (0.18-0.36)	0.96 (0.92-0.98)	0.68 (0.51-0.81)	2.6×	0.38
Skin	0.62 (0.57-0.66)	0.88 (0.86-0.90)	0.13 (0.08-0.20)	0.90 (0.88-0.92)	0.16 (0.10-0.24)	1.3×	0.26
Upper GI	0.86 (0.81-0.90)	0.98 (0.97-0.98)	0.61 (0.49-0.71)	0.93 (0.91-0.94)	0.34 (0.26-0.42)	6.1×	0.07
Urological	0.73 (0.68-0.79)	0.84 (0.81-0.87)	0.30 (0.22-0.40)	0.95 (0.92-0.97)	0.60 (0.46-0.73)	3.0×	0.44

Abbreviations: AUC, area-under-curve; GI, gastro intestinal; NPV, negative predictive value; PPV, positive predictive value.

**Figure 2:**
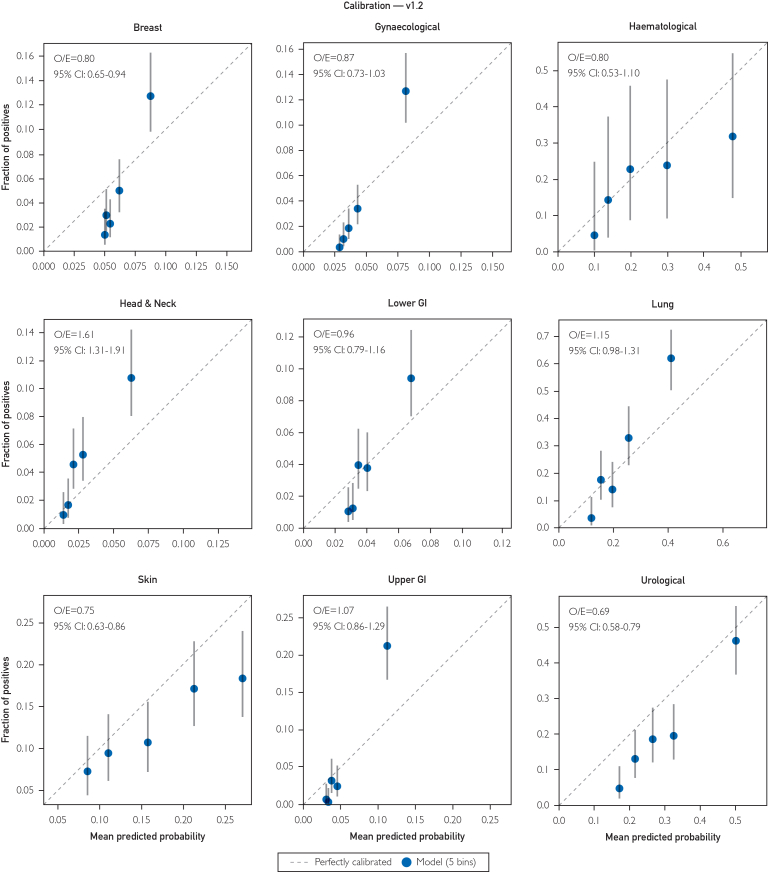
Plots of calibration curves per pathway for PinPoint Tests version 1.2, annotated with observed/expected (O/E) ratio. The dashed line indicates perfect calibration.

Calibration was also assessed. Figure 2 shows the calibration plots for each v1.2 PinPoint Test. These include 95% error bars and O/E ratios with 95% CIs. Calibration for most pathways was fair with respect to mean predicted risk, with O/E ratios consistent with 1 for 5 pathways. Predicted risks were lower than observed for head & neck (O/E 1.61, 95% CI, 1.31-1.91), and higher than observed for breast (0.80, 0.65-0.94), skin (0.75, 0.63-0.86), and urological (0.69, 0.58-0.79). Further information regarding calibration may be found in the Supplemental Appendix.

**Figure 3:**
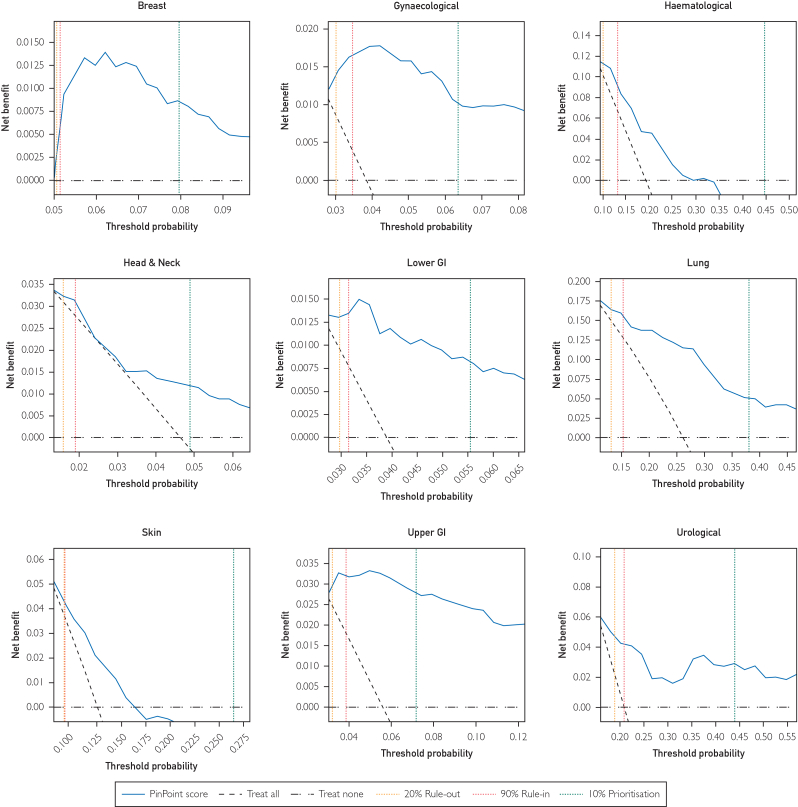
Decision curve analysis for each v1.2 PinPoint Test. Reference lines are included for the Treat All and Treat None strategies. The use case thresholds for each test are marked by dotted lines.

Decision Curve Analysis was performed to assess the net benefit of using the tests. Figure 3 shows the decision curves for each of the v1.2 tests. The thresholds corresponding to the three use cases are marked. For all pathways the test provides equal or higher net benefit than standard care at all displayed thresholds. More information is provided in Supplemental Appendix.

### Adverse Events

No adverse events were reported by the sites where blood was collected.

### Subgroup Analyses

Subgroup analyses stratified by sex, age, and ethnicity were performed using the primary analysis dataset. The results of these analyses can be found in the Supplemental Appendix. Ethnicity data was missing for a large proportion of patients in most pathways, and so conclusions cannot be drawn for the ethnicity analysis. For the age and sex analyses, no substantial differences were found across the various subgroups.

### Temporal Analyses

Eight pathways showed no statistically significant difference in AUC between the two halves. In the lung pathway, a modest drop in AUC is observed, corresponding to an observed increase in prevalence. This is covered in the Discussion section. For full results see the Supplemental Appendix.

### Performance of v1.1 Versus v1.2

Reduced v1.1 performance was observed in the interim analyses for the LGI, breast, skin, and urological pathways, caused by missing-not-at-random training values not being adequately accounted for by the gradient boosting models used in the algorithm. The v1.2 algorithms correct for this.

## Discussion

### Summary of Main Findings

This is the first large, prospective, real-world evaluation in NHS England of such a technology. It reports both the diagnostic accuracy of the tests and the practicality of deploying them in a health care setting. High-risk patients could be prioritized, expediting their onward clinical journey through the USC pathway, potentially leading to earlier cancer detection. For the lowest-risk patients, monitoring in primary care may be more appropriate than undergoing invasive diagnostic tests for cancer. The gynecologic, head and neck, lower GI, lung, and upper GI tests have performance that show potential clinical utility, and in several of these pathways there is currently no good early triage tool available; these tests could therefore address a pressing need.

Despite many pathways comprising a biologically heterogeneous mix of cancers, the tests still achieved good diagnostic accuracy.^9^, ^10^, ^11^, ^12^, ^13^, ^14^, ^15^ The consistently strong performance across such diverse pathologies suggests that the model is capturing shared signatures of malignancy rather than overfitting to any single tumor type.

One objective of the service evaluation was to assess the feasibility of deploying a test of this nature at scale within health service infrastructure. The evaluation demonstrated that such a deployment is achievable, particularly around challenges relating to integrating with pathology services and requesting systems, use of existing phlebotomy infrastructure, and engagement with clinicians across 5 secondary care Trusts and 170 General Practitioner (GP) surgeries.

Some pathways benefit from existing triage tools.

1. The urological test did not achieve a substantial improvement over prostate specific antigen. Prostate cancer dominates the urological pathway; there may be good sensitivity to other urological cancer types that larger or more targeted cohorts could uncover, and the addition of new analyte measurements could improve the test for prostate cancer.
2. FIT achieves AUC∼0.80 for colorectal cancer in the lower GI pathway,^12^ but 39% (36/93) of cancers in this cohort were non-colorectal. The PinPoint Test may complement FIT by covering non-colorectal cancers and FIT non-returners.
3. CA125 is used for some patients referred to the gynecological pathway. The AUC for CA125 as a single analyte on the gynecological pathway in this work is 0.71 (0.66-0.77), compared to 0.81 (0.77-0.85) for the PinPoint Test. While this is an imperfect comparison, it suggests that there may be a role here for the gynecological test.

The breast and skin tests did not achieve a substantial improvement over baseline predictors such as age. In the case of skin, this is due to a lack of strong signals in the panel of measured blood analytes. The breast test was impacted by a large amount of missing data in the training set; the service evaluation data show clear signals for breast cancer, which suggests that future versions of the breast test could improve on current performance. The performance of the hematological test is still unclear. The number of patients enrolled for this test was small and so the lack of statistical power means that it is difficult to make strong evidential statements.

For the ethnicity analysis, small subgroup sizes and incomplete data mean no conclusions on performance may be drawn. For the sex analysis, all pathways except for breast and gynecological show comparable performance for men and women. For the age analysis, comparable performance is also observed across age groups for all pathways. This is notable, as good performance in the younger age groups could assist in identifying cancers, which would otherwise be diagnosed later.

A modest temporal variation was found for AUC in the lung pathway. This may be caused by the new NHS lung screening program referring CT-detected asymptomatic lung cancer patients into the USC pathway. This is supported by an increase in both the observed lung pathway prevalence over time and the overall conversion rate for the local lung USC pathway in this period; see the Supplemental Appendix for details. This suggests that the temporal variation may be the result of an increased proportion of early-stage lung cancers (the NHS lung screening program is expected to find 47-86% early-stage lung cancers^13^)

Reduced v1.1 performance was observed in the interim analyses for the LGI, breast, skin, and urological pathway. The cause of this was missing-not-at-random training data, in particular for tumor markers. The impact of this was not seen in the original development and validation work,^3^ because the validation data shared the same issue. The v1.1 models use the presence or absence of a measured tumor marker as informative, with the built-in gradient boosting method for handling missing data unable to correct for this. This had 2 impacts, (1) to inflate predicted probability in this cohort, where all tumor markers are measured for all patients, and (2) to degrade AUC in some pathways. The v1.2 algorithms correct successfully for this, and were locked down before any of the other interim analyses were performed, and before any of the final analyses were performed. The temporal AUC analysis shows no significant increase in AUC for the chronological first half of the breast, skin, urological, LGI pathways (corresponding to the interim analyses), suggesting that no significant information leakage has occurred as the result of implementing this correction.

### Strengths of the Work

This work comprises a large-scale, prospective, observational, real-world NHS service evaluation of nine United Kingdom Conformity Assessed-marked Software as a Medical Device multi-cancer early detection blood tests. The patients were all referred to an NHS USC pathway for possible cancer diagnosis, and the blood draws, logistics, and test results were all performed as they would be for routine clinical practice. Patients were enrolled from multiple secondary care centers and included patients enrolled in both primary and secondary care settings. This matches the intended deployment setting, so both performance and operational logistics are representative of routine clinical practice.

This work provides important evidence that the tests are robust to a change in laboratory setting; while the algorithms were trained on data from a Siemens laboratory at Leeds Teaching Hospitals NHS Trust, the work presented in this paper was carried out at a Roche/Sysmex laboratory at MYTT. Robustness to such changes is encouraging for potential roll-out across the NHS.

Patients were recruited consecutively from a large, ethnically diverse area that included both urban and rural populations. Nationally aggregated ethnicity statistics for USC referrals are provided by NHS England.^9^ Because of a large proportion of missing data in the ethnicity data collected for this work, we cannot say definitively that there is concordance between the population and the sample ethnicity demographic characteristics. However, based on the data available, patients from a range of different ethnic backgrounds are represented.

### Limitations of the Work

The evaluation was observational. PinPoint Test results were calculated and returned to the regional hub laboratory for aggregation but were not used clinically.

A hub model was used to allow the blood measurements to be performed more efficiently, with all samples being sent to the MYTT laboratory. In routine clinical use, it may be that the tests would be deployed to each secondary care trust. However, the services used in the hub model are standard for the transport of biological specimens, and as such, the impact is expected to be minimal.

Referral criteria changed during the study period: NICE updates to FIT guidance for lower GI USC referrals (NG12 in 2020, updated 2021 and 2023; DG30 replaced by DG56),^10^, ^11^, ^12^ and smaller updates to prostate (2021) and myeloma (2025) referral criteria will have affected those pathways.

Staff were instructed to try to enroll every eligible person. Recruitment used multiple modalities (in-person at consultation, phone, Accurx text, and posters in clinical areas). The large number of centers precluded quantitative monitoring of the effectiveness of successive recruitment. Regular contact was maintained with the centers throughout recruitment, and clinician selection over participants is not believed to be a relevant source of bias.

Not all pathways reached 100 confirmed cancers due to differences in recruitment rates and funding limitations (see Table 1). This is reflected in the size of the CIs for these pathways.

The exclusion of 3,197 (19.4%) patients from the analysis for various reasons (see Figure 1) could be a source of bias for this work. A sensitivity analysis of a reasonable worst-case impact on AUC for each pathway suggests that, under the conservative assumption that the test has no discriminative power in the excluded patients, the expected decrease in AUC is at most 0.08 in the worst-affected pathway (urological), and by less than 0.06 in the others. Demographic characteristics of included and excluded patients are compared in the Supplemental Appendix.

### Implications for Policy and Practice

This evaluation provides large-scale, prospective validation of the PinPoint Tests in a real-world clinical setting. The performance observed suggests that the tests have the potential to substantially improve triage in multiple USC pathways. These improvements in triage could benefit the patient, the GP, and the system as a whole:

1. High-risk patients could be expedited through the system;
2. Lowest-risk patients could avoid unnecessary invasive medical testing for cancer;
3. A reduction in USC referrals could relieve pressure on downstream diagnostic services;
4. Optimizing clinic capacity could help Trusts achieve the national Faster Diagnosis Standard.

The Upper GI test, which has the strongest performance, addresses a high-volume (250,000 referrals/year), low-yield (∼3%) pathway with no current triage tool.

The Head & Neck test addresses another high-volume (285,000 referrals/year) pathway with no routine triage tool, where only one third of cancers are currently diagnosed early and only half start treatment within the NHS 62-day target.

The gynecological test (validated primarily against endometrial cancer, 61% of cohort cancers) could reduce demand for hysteroscopy, of which ∼100,000 are performed annually in England with a 5%-10% cancer yield.

This analysis covers patients in the USC pathways, but for the lung test the strong AUC result indicates a wider potential utility in spaces like the NHS Lung Health Check Programme. The test could offer enhanced rule-out and routes to safety netting, reducing pressures on lung computed tomography scanning.

## Potential Competing Interests

Drs Savage, Lloyd, Neal, Skinner, Sansom, Tully, Ferguson, and Duffy are employed by, and are shareholders or option holders in, PinPoint Data Science. Both the University of Leeds and Leeds Teaching Hospitals Trust have a royalty agreement with PinPoint Data Science; Prof Richard Neal is a named inventor in this royalty agreement.

## Ethics Statement

The design of the service evaluation was formally approved by the West Yorkshire Research and Development Team in the West Yorkshire Integrated Care Board. All patients gave recorded verbal consent, as per the requirement for this NHS service evaluation.

## Supplemental Online Material

Supplemental Material

STARD Checklist

STROBE Checklist
