# Supplementary material for: Real-World Validation of PinPoint Blood Tests in the NHS: Multivariable Machine Learning to Predict Cancer Risk in Primary Care Urgent Referrals
Source: Mayo Clin Proc Digit Health. 2026 Jun 10;4(3):100382. doi: 10.1016/j.mcpdig.2026.100382 (PMC13325911; doi:10.1016/j.mcpdig.2026.100382)
Supplement: Supplemental Material [file mmc1.docx]

Supplemental Appendix

Table of Contents

[Enrolment End Dates 1](#_Toc233198888)

[Model Predictors 2](#_Toc233198889)

[Sample Size Calculations 3](#_Toc233198890)

[Outcomes 4](#_Toc233198891)

[Test Performance Characteristics 8](#_Toc233198892)

[Confusion Matrices 11](#_Toc233198893)

[Calibration 17](#_Toc233198894)

[Decision Curve Analysis 18](#_Toc233198895)

[Temporal Analysis 18](#_Toc233198896)

[Exclusions from Analysis 21](#_Toc233198897)

[Subgroup Analyses 27](#_Toc233198898)

[Checklists 34](#_Toc233198899)

# Enrolment End Dates

Supplemental Table 1 shows the data collection end dates for each USC pathway in the service evaluation.

Supplemental Table 1: Data collection end date, by USC pathway.

| **Pathway** | **Data Collection End Date** |
| --- | --- |
| Breast | July 2023 |
| Gynaecological | July 2025 |
| Haematological | July 2025 |
| Head and Neck | April 2025 |
| Lower GI | July 2023 |
| Lung | July 2025 |
| Skin | July 2023 |
| Upper GI | July 2025 |
| Urological | February 2023 |

# Model Predictors

The predictors used by the PinPoint Test Suite are:

- Age
- Sex
- Haemoglobin
- White blood cell count
- Red blood cell count
- Neutrophil count
- Platelet count
- Mean cell volume (MCV)
- Lymphocytes count
- Monocytes count
- Basophil count
- Eosinophil count
- Mean corpuscular haemoglobin concentration (MCHC)
- Haematocrit
- Red cell distribution width (RDW)
- Calcium
- Phosphate
- Albumin
- Sodium
- Potassium
- Creatinine
- Urea
- Bilirubin
- Alanine aminotransferase (ALT)
- Alkaline phosphatase (ALP)
- C-reactive protein (CRP)
- CA125
- CA19-9
- Carcinoembryonic antigen (CEA)
- CA15-3
- Prostate specific antigen (PSA)
- Alpha fetoprotein (AFP)
- Human chorionic gonadotrophin (HCG)

All the blood analytes are used clinically within the NHS and so are accurate and reliable. Similarly, concordance of blood analytes between labs is expected to be acceptable, as all labs are UKAS-accredited and the blood analytes are routinely used.

Values for age and sex are submitted alongside the PinPoint Test request. Sex is reported as “Male” or “Female”, age is reported in years, and both values were derived from the EHR. All blood predictors are continuous and real-valued. At the limit of detection, inequalities were replaced with the value of the limit (e.g. “<5” becomes “5”).

# Sample Size Calculations

The sample size was selected to assess calibration with adequate precision across five prespecified risk strata. For a fixed number of cancers per subgroup, and assuming independent and identically-distributed Bernoulli trials, the number of non-cancer cases will therefore follow a negative binomial distribution. From this, we can compute analytically the expected 95% confidence interval (CI) for a given predicted cancer probability, under the null hypothesis that the predicted cancer probability is well-calibrated, using the inverse cumulative distribution function for the negative binomial distribution, ${CDF}_{k}^{-1}\left( x \right)$. As the quantity of interest is the probability of non-cancer diagnosis, $\frac{r}{r+k}$, the 95% confidence intervals may be calculated as:

$$CI= \left[ \frac{r}{r+{CDF}_{k}^{-1}\left( 0.025 \right)},\frac{r}{r+{CDF}_{k}^{-1}\left( 0.975 \right)} \right]$$

From these calculations, 20 cancer patients per subgroup is judged to be adequate. Therefore, 100 patients with cancer are required for each urgent suspected cancer referral pathway. As patients are enrolled prospectively and consecutively, all presenting patients who consented were enrolled, and enrolment ended when 100 patients per pathway completed their urgent suspected cancer referral with a cancer diagnosis. The expected number patients to be enrolled for each pathway were calculated in advance of the evaluation and are included in Supplemental Table 2.

Supplemental Table 2: Cancer prevalence and expected number of patients enrolled for each urgent suspected cancer referral pathway

| **Pathway** | **Expected cancer prevalence** | **Expected number of cases** |
| --- | --- | --- |
| Breast | 5.5% | 1818 |
| Gynaecological | 3.9% | 2564 |
| Haematological | 20.1% | 498 |
| Head and Neck | 2.8% | 3571 |
| Lower Gastrointestinal | 3.1% | 3226 |
| Lung | 15.3% | 654 |
| Skin | 6.5% | 1538 |
| Upper Gastrointestinal | 4.1% | 2439 |
| Urological | 15.7% | 637 |

# Outcomes

The number of patients with each cancer diagnosis in each pathway are listed in Supplemental Table 3 below as ICD10 codes. Empty cells indicate that this diagnosis did not occur on this pathway. Diagnoses that were not received by more than one patient on any one pathway are recorded in the “other” category.

Supplemental Table 3: Number of patients who received each cancer diagnosis, in the form of ICD10 codes, for each USC pathway.

| **ICD10** | **Breast** | **Gynaecological** | **Haematological** | **Head and Neck** | **Lower GI** | **Lung** | **Skin** | **Upper GI** | **Urological** |
| --- | --- | --- | --- | --- | --- | --- | --- | --- | --- |
| C01 |  |  |  | 3 |  |  |  |  |  |
| C02 |  |  |  | 4 |  |  |  |  |  |
| C07 |  |  |  | 3 |  |  |  |  |  |
| C09 |  |  |  | 16 |  |  |  |  |  |
| C10 |  |  | 1 | 2 |  |  |  |  |  |
| C13 |  |  |  | 3 |  |  |  |  |  |
| C15 |  |  |  | 2 |  | 1 |  | 41 |  |
| C16 |  |  |  |  | 2 |  |  | 6 |  |
| C17 |  | 1 |  |  | 3 |  |  |  |  |
| C18 |  |  | 1 |  | 30 |  |  | 5 | 2 |
| C19 |  |  |  |  | 3 |  |  |  |  |
| C20 |  |  |  |  | 24 |  |  |  |  |
| C21 |  |  |  |  | 3 |  |  |  |  |
| C22 |  | 1 |  |  |  |  |  | 6 |  |
| C25 |  |  | 1 |  | 1 |  |  | 12 |  |
| C32 |  |  |  | 13 |  |  |  |  |  |
| C34 |  | 1 |  | 1 | 5 | 91 |  | 3 |  |
| C38 |  |  |  |  |  | 2 |  |  |  |
| C43 |  |  |  |  | 1 |  | 17 |  |  |
| C44 |  |  |  | 4 | 2 | 1 | 93 |  |  |
| C45 |  |  |  |  |  | 4 |  |  |  |
| C48 |  | 8 |  | 1 | 1 |  |  |  |  |
| C50 | 100 | 1 | 1 |  | 1 |  | 1 |  |  |
| C51 |  | 9 |  |  |  |  |  |  |  |
| C52 |  | 5 |  |  |  |  |  |  |  |
| C53 |  | 4 |  |  |  |  |  |  |  |
| C54 |  | 69 |  |  |  |  |  |  |  |
| C55 |  | 2 |  |  |  |  |  |  |  |
| C56 |  | 9 |  |  | 1 |  |  |  |  |
| C61 |  |  |  | 1 | 5 |  | 2 |  | 91 |
| C64 |  | 1 |  |  | 1 |  |  |  | 5 |
| C67 |  |  |  | 1 |  |  |  |  | 9 |
| C73 |  |  |  | 11 | 1 |  |  |  |  |
| C77 | 1 |  |  | 7 | 1 |  |  |  |  |
| C80 |  |  | 1 | 1 | 1 | 2 |  | 1 |  |
| C81 |  |  | 1 | 5 | 1 |  |  |  |  |
| C82 |  | 1 | 2 | 3 | 1 |  |  | 1 |  |
| C83 |  |  | 2 | 7 |  | 1 | 1 | 2 |  |
| C90 |  |  | 6 | 1 | 2 |  |  |  |  |
| C91 |  |  | 2 | 1 |  |  |  |  |  |
| D03 |  |  |  |  |  |  | 10 |  |  |
| D04 |  |  |  |  |  |  | 23 |  |  |
| D05 | 6 |  |  |  |  |  |  |  |  |
| Other | 0 | 2 | 3 | 7 | 3 | 1 | 0 | 2 | 3 |

Supplemental Table 4 shows the non-cancer diagnoses, as ICD10 codes, that were recorded for patients who were not diagnosed with cancer. This list of diagnoses is not exhaustive. Patients whose referral was completed but a non-cancer diagnosis was not reported are included in the “Not reported” group. Empty cells indicate that this diagnosis did not occur on this pathway. Diagnoses that were not received by more than one patient on any one pathway are recorded in the “other” category.

Supplemental Table 4: Number of patients who received each non-cancer diagnosis, in the form of ICD10 codes, for each USC pathway.

| **ICD10** | **Breast** | **Gynaecological** | **Haematological** | **Head and Neck** | **Lower GI** | **Lung** | **Skin** | **Upper GI** | **Urological** |
| --- | --- | --- | --- | --- | --- | --- | --- | --- | --- |
| B07 |  |  |  |  |  |  | 4 |  |  |
| D10 |  |  |  | 35 |  |  |  |  |  |
| D11 |  |  |  | 5 |  |  |  |  |  |
| D12 |  |  |  |  | 78 | 3 |  |  |  |
| D13 |  |  |  |  |  |  |  | 31 |  |
| D14 |  |  |  | 45 |  | 14 |  |  |  |
| D18 |  |  |  |  |  |  | 3 |  |  |
| D22 |  |  |  |  |  |  | 20 |  |  |
| D23 |  |  |  |  |  |  | 13 |  |  |
| D24 | 468 |  |  |  |  |  |  |  |  |
| D26 |  | 101 |  |  |  |  |  |  |  |
| D27 |  | 4 |  |  |  |  |  |  |  |
| D28 |  | 13 |  |  |  |  |  |  |  |
| D34 |  |  |  | 7 |  |  |  |  |  |
| D36 |  |  |  | 5 |  |  |  |  |  |
| D39 |  | 2 |  |  |  |  |  |  |  |
| D40 |  |  |  |  |  |  |  |  | 6 |
| D41 |  | 1 |  |  | 1 |  |  |  | 8 |
| D47 |  |  | 6 |  |  |  |  |  |  |
| D48 |  |  |  |  |  |  | 2 |  |  |
| D75 |  |  | 2 |  |  |  |  |  |  |
| I89 |  |  | 4 | 2 |  |  |  |  |  |
| J38 |  |  |  | 238 |  |  |  |  |  |
| J98 |  |  |  |  |  | 25 |  |  |  |
| K09 |  |  |  | 18 |  |  |  |  |  |
| K11 |  |  |  | 2 |  |  |  |  |  |
| K13 |  |  |  | 2 |  |  |  |  |  |
| K14 |  |  |  | 2 |  |  |  |  |  |
| K20 |  |  |  | 2 |  |  |  | 5 |  |
| K22 |  |  |  |  |  |  |  | 145 |  |
| K29 |  |  |  |  |  |  |  | 16 |  |
| K62 |  |  |  |  | 2 |  |  |  |  |
| K63 |  |  | 1 |  | 184 |  |  |  |  |
| L57 |  |  |  |  |  |  | 21 |  |  |
| L82 |  |  |  |  |  |  | 29 |  |  |
| L98 |  |  |  |  |  |  | 81 |  |  |
| N32 |  |  |  |  | 2 |  |  |  | 14 |
| N42 |  |  |  |  |  |  |  |  | 53 |
| N62 | 6 |  |  |  |  |  |  |  |  |
| N64 | 59 |  |  |  |  |  |  |  |  |
| N83 |  | 4 |  |  |  |  |  |  |  |
| N85 |  | 142 |  |  |  |  |  |  |  |
| N88 |  | 64 |  |  |  |  |  |  |  |
| N89 |  | 7 |  |  |  |  |  |  |  |
| N90 |  | 12 |  |  |  |  |  |  |  |
| Other | 1 | 1 | 1 | 2 | 3 | 0 | 2 | 3 | 3 |
| Not reported | 1558 | 2488 | 73 | 1629 | 2025 | 249 | 845 | 1135 | 347 |

# Test Performance Characteristics

Supplemental Tables 5, 6, and 7 provide the performance characteristics for the PinPoint Test suite version 1.1 tests. Each table has thresholds chosen to meet the use-case aims.

Supplemental Table 5: Test validation set performance characteristics for PinPoint Test version 1.1. Aim: 20% rule-out.

| **Pathway** | **AUC**  **(95% CI)** | **NPV**  **(95% CI)** | **Sensitivity**  **(95% CI)** | **Specificity**  **(95% CI)** | **PPV**  **(95% CI)** | **Threshold** |
| --- | --- | --- | --- | --- | --- | --- |
| Breast | 0.57 (0.51–0.62) | 0.96 (0.93–0.97) | 0.83 (0.74–0.89) | 0.20 (0.18–0.22) | 0.05 (0.04–0.06) | 0.26 |
| Gynaecological | 0.69 (0.63–0.74) | 0.99 (0.98–1.00) | 0.95 (0.88–0.98) | 0.20 (0.18–0.21) | 0.05 (0.04–0.05) | 0.19 |
| Haematological | 0.52 (0.37–0.67) | 0.77 (0.54–0.91) | 0.76 (0.52–0.91) | 0.20 (0.12–0.30) | 0.19 (0.11–0.29) | 0.57 |
| Head and Neck | 0.53 (0.47–0.59) | 0.96 (0.94–0.98) | 0.85 (0.75–0.91) | 0.20 (0.18–0.22) | 0.05 (0.04–0.06) | 0.24 |
| Lower GI | 0.65 (0.60–0.71) | 0.99 (0.98–1.00) | 0.96 (0.89–0.99) | 0.20 (0.18–0.22) | 0.05 (0.04–0.06) | 0.19 |
| Lung | 0.66 (0.60–0.73) | 0.84 (0.73–0.91) | 0.89 (0.81–0.94) | 0.20 (0.16–0.25) | 0.28 (0.24–0.34) | 0.46 |
| Skin | 0.53 (0.48–0.58) | 0.90 (0.85–0.93) | 0.84 (0.77–0.90) | 0.20 (0.17–0.22) | 0.13 (0.11–0.16) | 0.51 |
| Upper GI | 0.80 (0.75–0.86) | 0.99 (0.96–1.00) | 0.96 (0.89–0.99) | 0.20 (0.18–0.22) | 0.07 (0.05–0.08) | 0.20 |
| Urological | 0.62 (0.56–0.68) | 0.87 (0.78–0.93) | 0.88 (0.80–0.93) | 0.20 (0.16–0.24) | 0.22 (0.18–0.26) | 0.35 |

Supplemental Table 6: Test validation set performance characteristics for PinPoint Test version 1.1. Aim: 90% rule-in.

| **Pathway** | **AUC**  **(95% CI)** | **NPV**  **(95% CI)** | **Sensitivity**  **(95% CI)** | **Specificity**  **(95% CI)** | **PPV**  **(95% CI)** | **Threshold** |
| --- | --- | --- | --- | --- | --- | --- |
| Breast | 0.57 (0.51–0.62) | 0.97 (0.95–0.98) | 0.90 (0.82–0.95) | 0.17 (0.15–0.19) | 0.05 (0.04–0.06) | 0.24 |
| Gynaecological | 0.69 (0.63–0.74) | 0.99 (0.97–0.99) | 0.89 (0.82–0.94) | 0.29 (0.28–0.31) | 0.05 (0.04–0.06) | 0.22 |
| Haematological | 0.52 (0.37–0.67) | 0.84 (0.60–0.96) | 0.86 (0.63–0.96) | 0.18 (0.11–0.28) | 0.20 (0.13–0.30) | 0.56 |
| Head and Neck | 0.53 (0.47–0.59) | 0.96 (0.93–0.98) | 0.90 (0.81–0.95) | 0.12 (0.11–0.14) | 0.05 (0.04–0.06) | 0.20 |
| Lower GI | 0.65 (0.60–0.71) | 0.99 (0.97–0.99) | 0.89 (0.81–0.94) | 0.30 (0.28–0.32) | 0.05 (0.04–0.06) | 0.23 |
| Lung | 0.66 (0.60–0.73) | 0.84 (0.73–0.91) | 0.89 (0.81–0.94) | 0.20 (0.16–0.25) | 0.28 (0.24–0.34) | 0.46 |
| Skin | 0.53 (0.48–0.58) | 0.91 (0.85–0.94) | 0.90 (0.83–0.94) | 0.14 (0.12–0.17) | 0.13 (0.11–0.15) | 0.48 |
| Upper GI | 0.80 (0.75–0.86) | 0.99 (0.97–0.99) | 0.90 (0.81–0.95) | 0.51 (0.49–0.54) | 0.10 (0.08–0.12) | 0.30 |
| Urological | 0.62 (0.56–0.68) | 0.88 (0.79–0.93) | 0.89 (0.81–0.94) | 0.20 (0.16–0.24) | 0.22 (0.18–0.26) | 0.35 |

Supplemental Table 7: Test validation set performance characteristics for PinPoint Test version 1.1. Aim: 10% prioritisation.

| **Pathway** | **AUC**  **(95% CI)** | **NPV**  **(95% CI)** | **Sensitivity**  **(95% CI)** | **Specificity**  **(95% CI)** | **PPV**  **(95% CI)** | **Threshold** |
| --- | --- | --- | --- | --- | --- | --- |
| Breast | 0.57 (0.51–0.62) | 0.95 (0.94–0.96) | 0.12 (0.07–0.20) | 0.90 (0.89–0.91) | 0.06 (0.03–0.10) | 0.71 |
| Gynaecological | 0.69 (0.63–0.74) | 0.97 (0.96–0.98) | 0.34 (0.26–0.44) | 0.91 (0.90–0.92) | 0.13 (0.10–0.18) | 0.36 |
| Haematological | 0.52 (0.37–0.67) | 0.81 (0.72–0.88) | 0.14 (0.04–0.37) | 0.91 (0.82–0.96) | 0.27 (0.07–0.61) | 0.93 |
| Head and Neck | 0.53 (0.47–0.59) | 0.95 (0.94–0.96) | 0.09 (0.05–0.17) | 0.89 (0.88–0.91) | 0.04 (0.02–0.08) | 0.42 |
| Lower GI | 0.65 (0.60–0.71) | 0.97 (0.96–0.98) | 0.28 (0.19–0.38) | 0.91 (0.89–0.92) | 0.11 (0.07–0.15) | 0.47 |
| Lung | 0.66 (0.60–0.73) | 0.77 (0.72–0.81) | 0.19 (0.13–0.29) | 0.93 (0.89–0.96) | 0.50 (0.34–0.66) | 0.66 |
| Skin | 0.53 (0.48–0.58) | 0.88 (0.86–0.90) | 0.14 (0.09–0.21) | 0.90 (0.88–0.92) | 0.17 (0.11–0.25) | 0.63 |
| Upper GI | 0.80 (0.75–0.86) | 0.97 (0.95–0.97) | 0.44 (0.33–0.56) | 0.92 (0.90–0.93) | 0.24 (0.18–0.32) | 0.44 |
| Urological | 0.62 (0.56–0.68) | 0.83 (0.79–0.86) | 0.25 (0.17–0.34) | 0.93 (0.90–0.95) | 0.48 (0.35–0.62) | 0.68 |

# Confusion Matrices

Supplemental Tables 8, 9, and 10 show the cross-tabulation of the PinPoint Test results for version 1.1 and cancer diagnoses for each urgent suspected cancer pathway. For each pathway and aim, the thresholds used were chosen to fulfil the aim. The thresholds were the same as those used in Supplemental Tables 5, 6, and 7.

Supplemental Tables 11, 12, and 13 show the cross-tabulation for the PinPoint Test version 1.2. The thresholds used were the same as those used in Tables 2–4 in the main paper.

Supplemental Table 8: Cross-tabulation of PinPoint Test v1.1 results and cancer diagnoses. Aim: 20% rule-out.

| **Pathway** | **Outcome** | **Predicted Negative** | **Predicted Positive** |
| --- | --- | --- | --- |
| Breast |  |  |  |
|  | Non-cancer | 417 | 1675 |
|  | Cancer | 18 | 89 |
| Gynaecological |  |  |  |
|  | Non-cancer | 562 | 2277 |
|  | Cancer | 6 | 108 |
| Haematological |  |  |  |
|  | Non-cancer | 17 | 70 |
|  | Cancer | 5 | 16 |
| Head and Neck |  |  |  |
|  | Non-cancer | 397 | 1597 |
|  | Cancer | 15 | 82 |
| Lower GI |  |  |  |
|  | Non-cancer | 458 | 1837 |
|  | Cancer | 4 | 89 |
| Lung |  |  |  |
|  | Non-cancer | 58 | 233 |
|  | Cancer | 11 | 92 |
| Skin |  |  |  |
|  | Non-cancer | 202 | 818 |
|  | Cancer | 23 | 124 |
| Upper GI |  |  |  |
|  | Non-cancer | 265 | 1070 |
|  | Cancer | 3 | 76 |
| Urological |  |  |  |
|  | Non-cancer | 86 | 345 |
|  | Cancer | 13 | 97 |

Supplemental Table 9: Cross-tabulation of PinPoint Test v1.1 results and cancer diagnoses. Aim: 90% rule-in.

| **Pathway** | **Outcome** | **Predicted Negative** | **Predicted Positive** |
| --- | --- | --- | --- |
| Breast |  |  |  |
|  | Non-cancer | 354 | 1738 |
|  | Cancer | 11 | 96 |
| Gynaecological |  |  |  |
|  | Non-cancer | 832 | 2007 |
|  | Cancer | 12 | 102 |
| Haematological |  |  |  |
|  | Non-cancer | 16 | 71 |
|  | Cancer | 3 | 18 |
| Head and Neck |  |  |  |
|  | Non-cancer | 249 | 1745 |
|  | Cancer | 10 | 87 |
| Lower GI |  |  |  |
|  | Non-cancer | 689 | 1606 |
|  | Cancer | 10 | 83 |
| Lung |  |  |  |
|  | Non-cancer | 58 | 233 |
|  | Cancer | 11 | 92 |
| Skin |  |  |  |
|  | Non-cancer | 147 | 873 |
|  | Cancer | 15 | 132 |
| Upper GI |  |  |  |
|  | Non-cancer | 684 | 651 |
|  | Cancer | 8 | 71 |
| Urological |  |  |  |
|  | Non-cancer | 85 | 346 |
|  | Cancer | 12 | 98 |

Supplemental Table 10: Cross-tabulation of PinPoint Test v1.1 results and cancer diagnoses. Aim: 10% prioritisation.

| **Pathway** | **Outcome** | **Predicted Negative** | **Predicted Positive** |
| --- | --- | --- | --- |
| Breast |  |  |  |
|  | Non-cancer | 1885 | 207 |
|  | Cancer | 94 | 13 |
| Gynaecological |  |  |  |
|  | Non-cancer | 2582 | 257 |
|  | Cancer | 75 | 39 |
| Haematological |  |  |  |
|  | Non-cancer | 79 | 8 |
|  | Cancer | 18 | 3 |
| Head and Neck |  |  |  |
|  | Non-cancer | 1779 | 215 |
|  | Cancer | 88 | 9 |
| Lower GI |  |  |  |
|  | Non-cancer | 2078 | 217 |
|  | Cancer | 67 | 26 |
| Lung |  |  |  |
|  | Non-cancer | 271 | 20 |
|  | Cancer | 83 | 20 |
| Skin |  |  |  |
|  | Non-cancer | 917 | 103 |
|  | Cancer | 126 | 21 |
| Upper GI |  |  |  |
|  | Non-cancer | 1226 | 109 |
|  | Cancer | 44 | 35 |
| Urological |  |  |  |
|  | Non-cancer | 402 | 29 |
|  | Cancer | 83 | 27 |

Supplemental Table 11: Cross-tabulation of PinPoint Test v1.2 results and cancer diagnoses. Aim: 20% rule-out.

| **Pathway** | **Outcome** | **Predicted Negative** | **Predicted Positive** |
| --- | --- | --- | --- |
| Breast |  |  |  |
|  | Non-cancer | 418 | 1674 |
|  | Cancer | 6 | 101 |
| Gynaecological |  |  |  |
|  | Non-cancer | 567 | 2272 |
|  | Cancer | 1 | 113 |
| Haematological |  |  |  |
|  | Non-cancer | 17 | 70 |
|  | Cancer | 0 | 21 |
| Head and Neck |  |  |  |
|  | Non-cancer | 398 | 1596 |
|  | Cancer | 4 | 93 |
| Lower GI |  |  |  |
|  | Non-cancer | 458 | 1837 |
|  | Cancer | 5 | 88 |
| Lung |  |  |  |
|  | Non-cancer | 58 | 233 |
|  | Cancer | 3 | 100 |
| Skin |  |  |  |
|  | Non-cancer | 203 | 817 |
|  | Cancer | 14 | 133 |
| Upper GI |  |  |  |
|  | Non-cancer | 266 | 1069 |
|  | Cancer | 2 | 77 |
| Urological |  |  |  |
|  | Non-cancer | 86 | 345 |
|  | Cancer | 4 | 106 |

Supplemental Table 12: Cross-tabulation of PinPoint Test v1.2 results and cancer diagnoses. Aim: 90% rule-in.

| **Pathway** | **Outcome** | **Predicted Negative** | **Predicted Positive** |
| --- | --- | --- | --- |
| Breast |  |  |  |
|  | Non-cancer | 678 | 1414 |
|  | Cancer | 11 | 96 |
| Gynaecological |  |  |  |
|  | Non-cancer | 1359 | 1480 |
|  | Cancer | 12 | 102 |
| Haematological |  |  |  |
|  | Non-cancer | 37 | 50 |
|  | Cancer | 3 | 18 |
| Head and Neck |  |  |  |
|  | Non-cancer | 809 | 1185 |
|  | Cancer | 10 | 87 |
| Lower GI |  |  |  |
|  | Non-cancer | 749 | 1546 |
|  | Cancer | 10 | 83 |
| Lung |  |  |  |
|  | Non-cancer | 106 | 185 |
|  | Cancer | 11 | 92 |
| Skin |  |  |  |
|  | Non-cancer | 211 | 809 |
|  | Cancer | 15 | 132 |
| Upper GI |  |  |  |
|  | Non-cancer | 743 | 592 |
|  | Cancer | 8 | 71 |
| Urological |  |  |  |
|  | Non-cancer | 149 | 282 |
|  | Cancer | 12 | 98 |

Supplemental Table 13: Cross-tabulation of PinPoint Test v1.2 results and cancer diagnoses. Aim: 10% prioritisation.

| **Pathway** | **Outcome** | **Predicted Negative** | **Predicted Positive** |
| --- | --- | --- | --- |
| Breast |  |  |  |
|  | Non-cancer | 1907 | 185 |
|  | Cancer | 72 | 35 |
| Gynaecological |  |  |  |
|  | Non-cancer | 2590 | 249 |
|  | Cancer | 67 | 47 |
| Haematological |  |  |  |
|  | Non-cancer | 78 | 9 |
|  | Cancer | 19 | 2 |
| Head and Neck |  |  |  |
|  | Non-cancer | 1817 | 177 |
|  | Cancer | 64 | 33 |
| Lower GI |  |  |  |
|  | Non-cancer | 2088 | 207 |
|  | Cancer | 61 | 32 |
| Lung |  |  |  |
|  | Non-cancer | 278 | 13 |
|  | Cancer | 76 | 27 |
| Skin |  |  |  |
|  | Non-cancer | 922 | 98 |
|  | Cancer | 128 | 19 |
| Upper GI |  |  |  |
|  | Non-cancer | 1241 | 94 |
|  | Cancer | 31 | 48 |
| Urological |  |  |  |
|  | Non-cancer | 409 | 22 |
|  | Cancer | 77 | 33 |

# Calibration

Supplemental Figure 1 and Figure 2 (main paper) show calibration curves for predictions by the algorithms for each pathway, calculated using equal occupancy bins. Good calibration means that the algorithm results can be interpreted as being the probability of a given patient having cancer and is indicated by the points lying along the dashed diagonal line. The error bars show the 95% binomial proportion confidence interval, calculated using the Wilson score with continuity correction. For each pathway the observed-to-expected (O/E) ratio is shown with a bootstrapped 95% confidence interval. Supplemental Figure 1 shows the calibration for the version 1.1 PinPoint Tests.


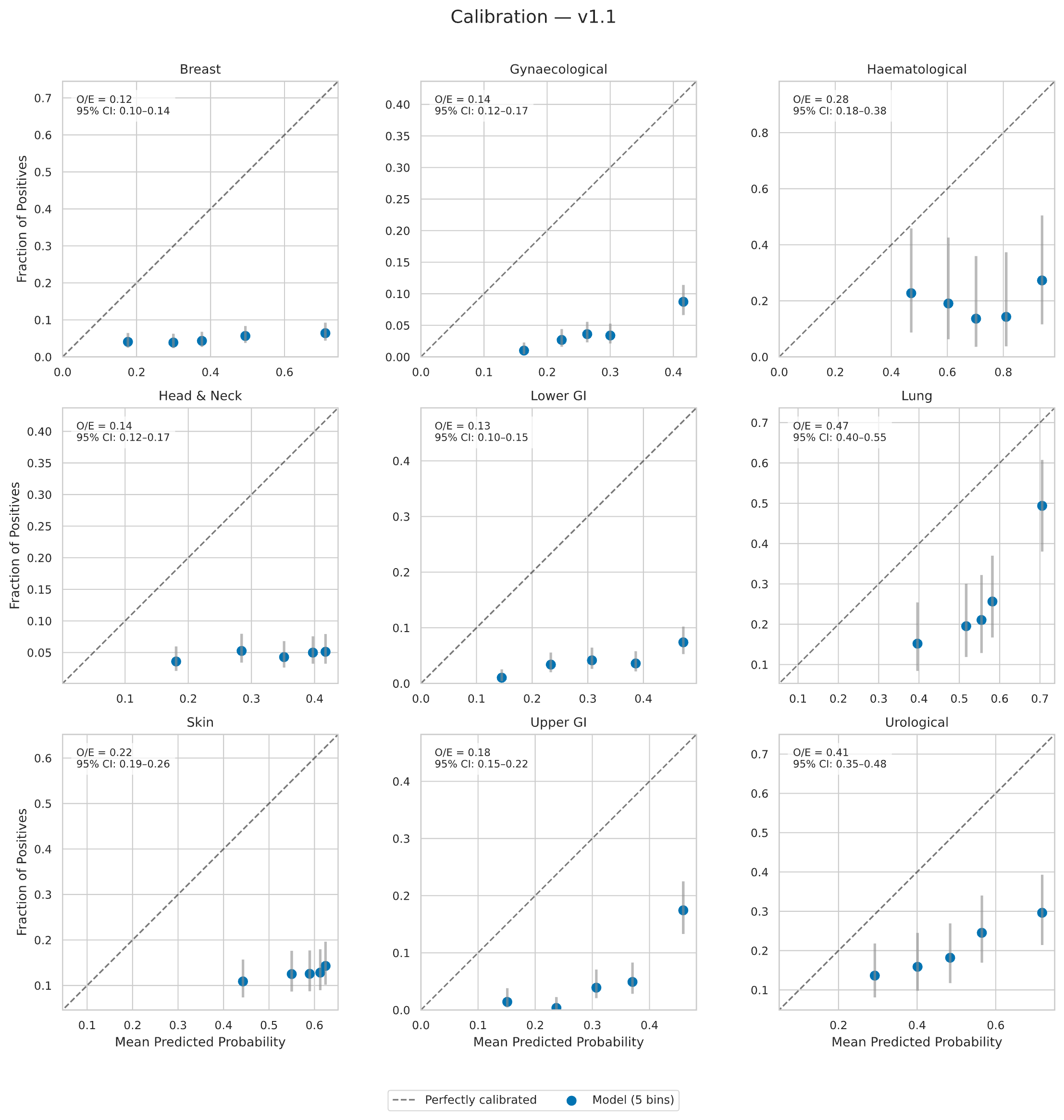


Supplemental Figure 1: Plots of calibration curves per pathway for PinPoint Tests version 1.1, annotated with observed/expected (O/E) ratio. The dashed line indicates perfect calibration.

# Decision Curve Analysis

Decision curve analysis was performed using the python package dcurves.

For this analysis, the action is whether a patient on a USC pathway is prioritised for further investigation. Treat All would be the equivalent of standard care, where all patients meeting the referral criteria are referred. Treat None would not be clinically realistic but is provided for reference.

Due to the poor calibration of v1.1, decision curve analysis was not feasible and so these plots are not provided.

# Temporal Analysis

It was considered that factors such as the COVID-19 pandemic and changes to referral criteria could have impacted the population from which patients were recruited for the service evaluation, and hence the performance of the PinPoint Tests. Cancer prevalence and test AUC over time were therefore analysed.


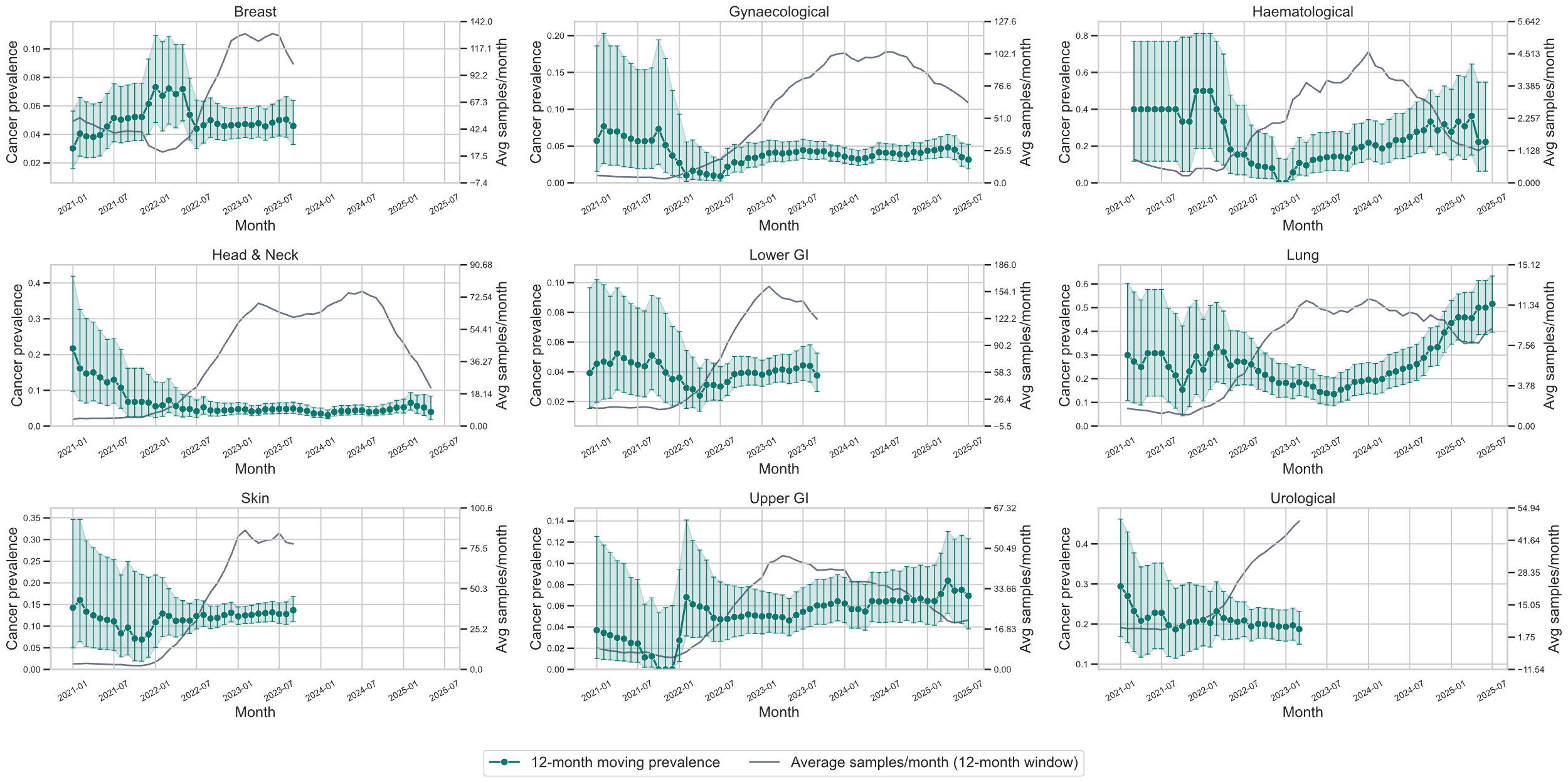


Supplemental Figure 2: Prevalence (moving average of 12 months) and average number of patients recruited per month (moving average of 12 months) for the recruitment period, for each USC pathway. 95% confidence intervals are marked for prevalence.

Supplemental Figure 2 shows the number of patients recruited and cancer prevalence as a rolling average over the recruitment time for each pathway. It may be observed that for all pathways the rate of recruitment of patients was low during the early part of the pandemic. The error bars on the prevalence for the initial months are very large, due to the smaller number of patients being recruited, and stabilise as the number of patients increased.


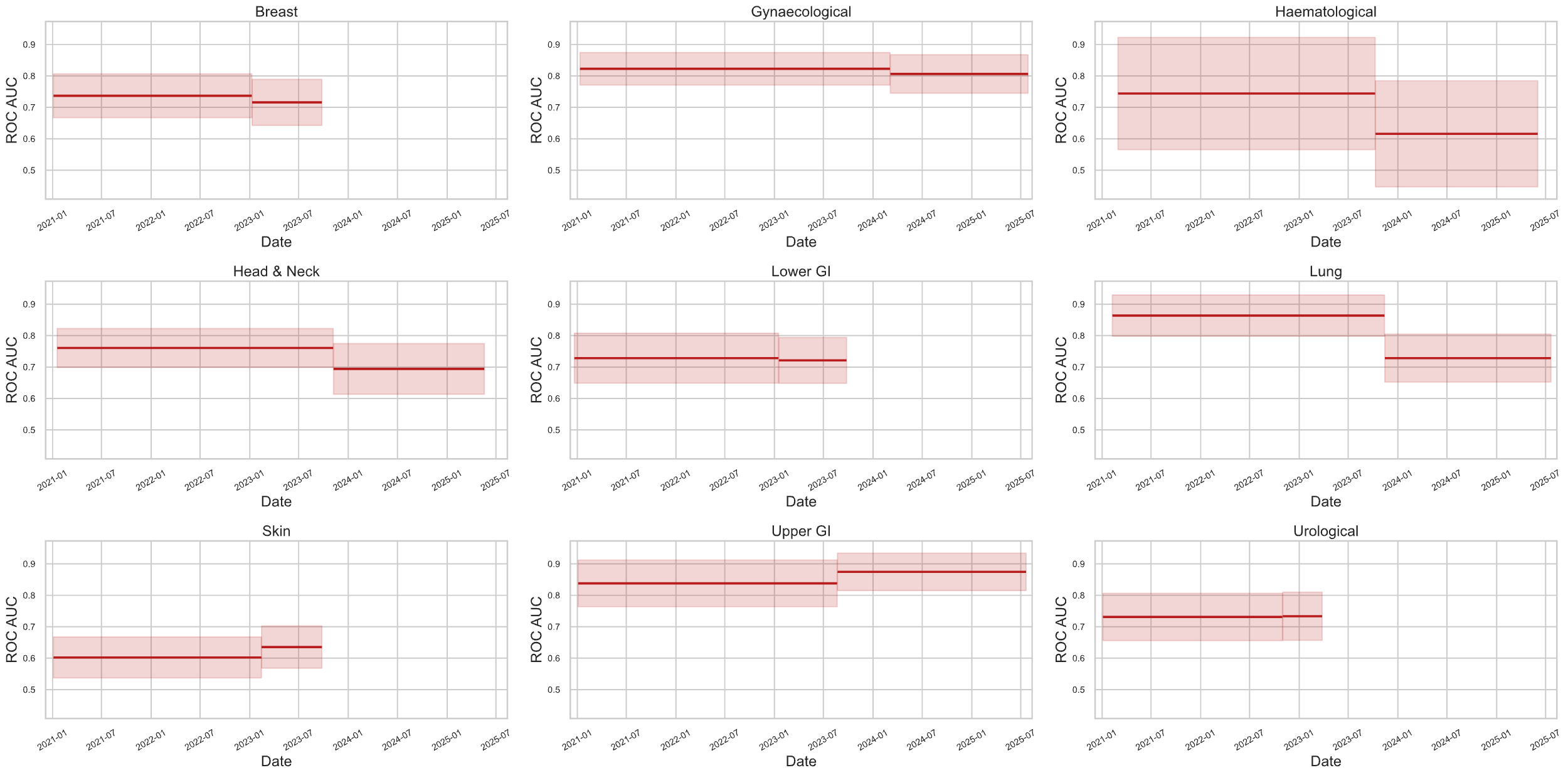


Supplemental Figure 3: AUC and 95% confidence intervals for each v1.2 PinPoint Test, with patients split into two equal sized bins by recruitment date.

Supplemental Figure 3 shows the AUC for each v1.2 test, with 95% confidence intervals, calculated by splitting the recruitment period for each test into two bins containing approximately equal numbers of patients.

DeLong’s test was used to test the difference between the AUC for the two periods for each test. Supplemental Table 14 shows the results of these tests.

Supplemental Table 14: PinPoint Test v1.2 AUCs for the two time periods when the recruitment period is split into two sections with equal numbers of patients. P values are the result of Delong’s test to compare ROC AUCs.

| **Pathway** | **AUC period 1 (95% CI)** | **AUC period 2 (95% CI)** | **P-value** |
| --- | --- | --- | --- |
| Breast | 0.74 (0.67–0.81) | 0.72 (0.64–0.79) | 0.67 |
| Gynaecological | 0.82 (0.77–0.87) | 0.81 (0.75–0.87) | 0.69 |
| Haematological | 0.74 (0.57–0.92) | 0.62 (0.45–0.78) | 0.31 |
| Head and neck | 0.76 (0.70–0.82) | 0.69 (0.61–0.77) | 0.20 |
| Lower GI | 0.73 (0.65–0.81) | 0.72 (0.65–0.79) | 0.90 |
| Lung | 0.86 (0.80–0.93) | 0.73 (0.65–0.80) | 0.0084 |
| Skin | 0.60 (0.54–0.67) | 0.64 (0.57–0.70) | 0.49 |
| Upper GI | 0.84 (0.76–0.91) | 0.87 (0.82–0.93) | 0.45 |
| Urological | 0.73 (0.66–0.81) | 0.73 (0.66–0.81) | 0.97 |


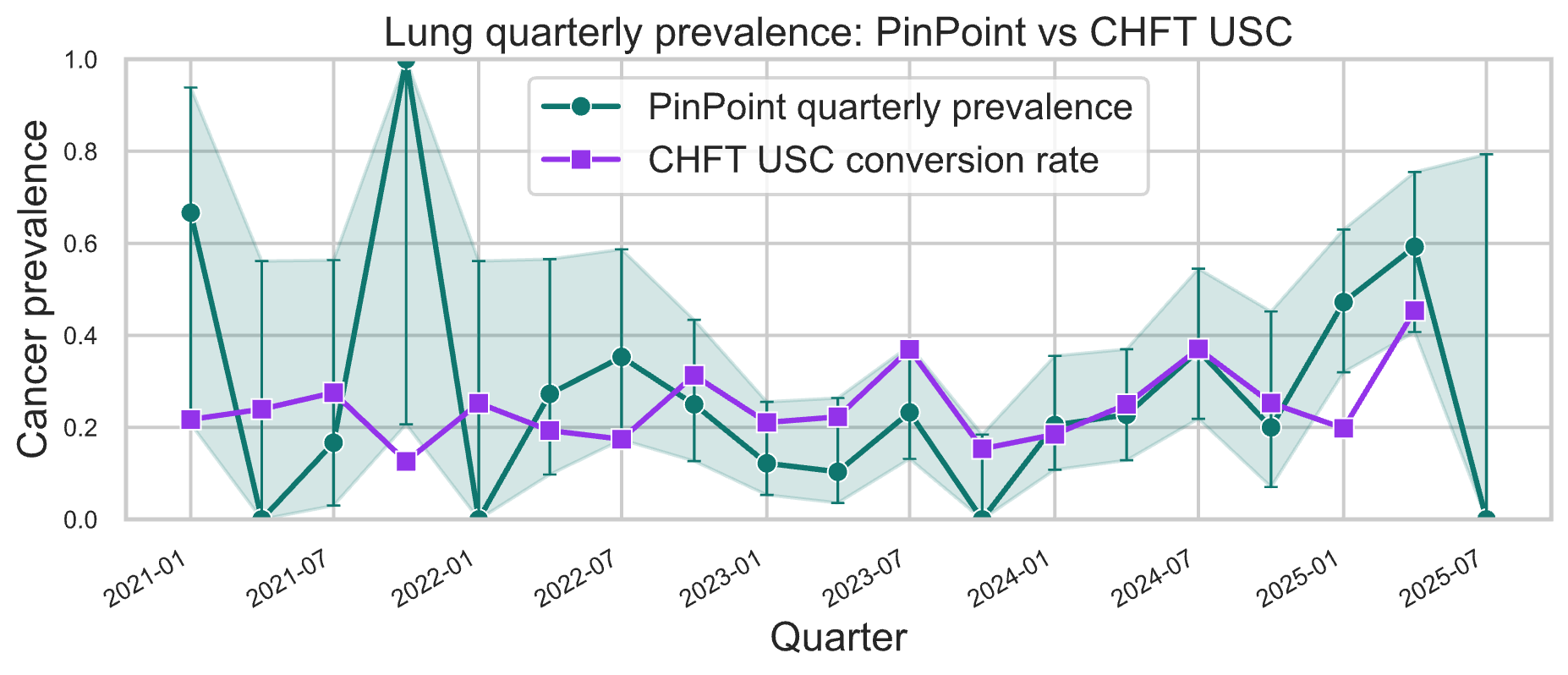


Supplemental Figure 4: Quarterly prevalence for the Lung pathway, showing the observed prevalence in the data presented here and the conversion rate for the Lung pathway at CHFT.

Eight pathways showed no statistically significant difference in AUC between the two halves. In the lung pathway, a modest drop in AUC is observed, corresponding to an increase in prevalence. The NHS has been piloting and deploying a new CT-based lung screening programme (including in the West Yorkshire region), the test-positive patients from which receive USC referrals; it is therefore possible that the observed increase in prevalence is caused by this. As this population is expected to be 47-86% early-stage cancers, a modest drop in AUC of this nature might be expected. A comparison between the quarterly CHFT Lung pathway conversion rate and the quarterly observed prevalence in the data presented here can be seen in Supplemental Figure 4.

For pathways other than lung, the changes to referral criteria described in the Discussion section did not result in statistically significant changes in performance.

# Exclusions from Analysis

To assess the impact of the exclusions from the analysis listed in Figure 1, the data that are available for the excluded patients were investigated.


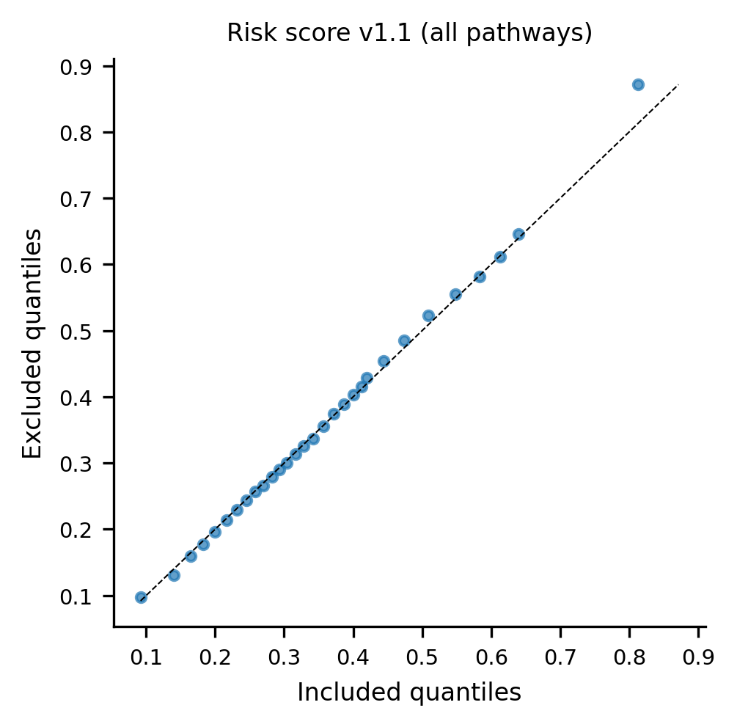


Supplemental Figure 5: PinPoint Test v1.1 results for patients excluded from the analysis for whom a result was available.


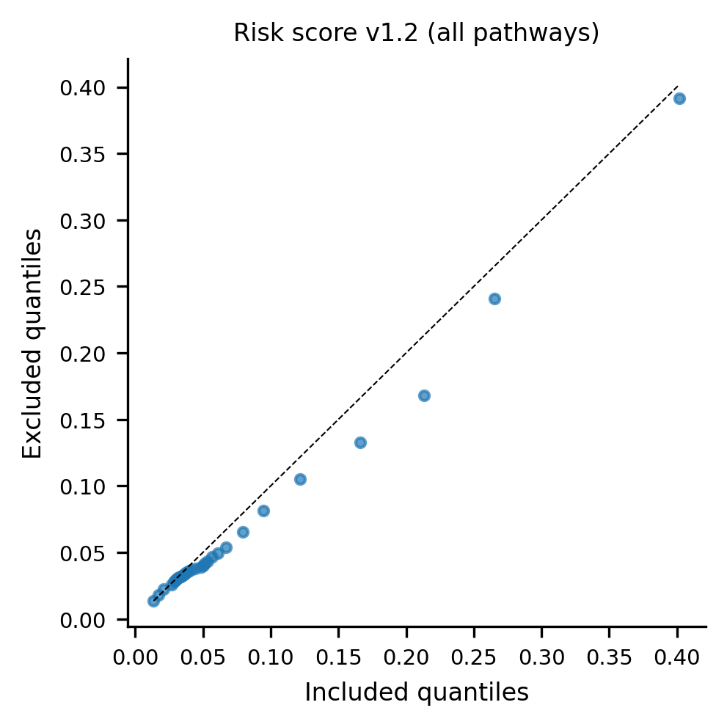


Supplemental Figure 6: PinPoint Test v1.2 results for patients excluded from the analysis for whom a result was available.

Supplemental Figure 5 and Supplemental Figure 6 show Q-Q plots of the v1.1 and v1.2 PinPoint Test results for the excluded patients for whom the data are available. Supplemental Figure 7 shows Q-Q plots of the analyte values for patients excluded from the analysis for whom the analyte values were available.


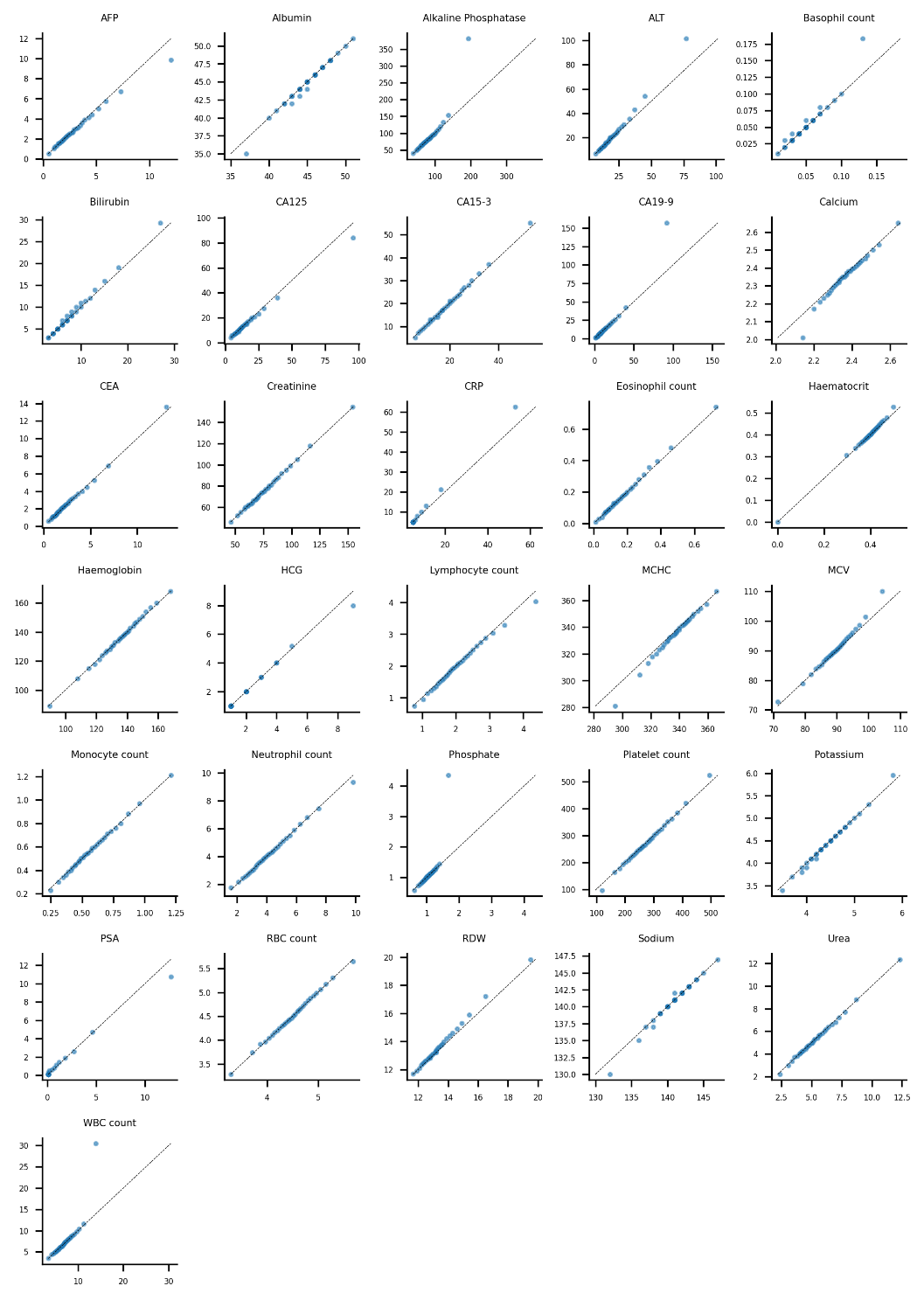


Supplemental Figure 7: Analyte values for patients excluded from the analysis for whom a result was available.

Supplemental Table 15 shows the breakdown of available and missing demographic information for the excluded patients, compared to the included patients. Demographic data were available for 1,209 of the 3,197 excluded patients (38%); some patients, such as those excluded because their data could not be extracted from NHS systems, had no demographic information captured.

Supplemental Table 15: Demographic information for patients included and excluded from the analysis, for each pathway, where demographic data was available.

| **Group** | **Variable** | **Breast** | **Gynaecological** | **Haematological** | **Head & Neck** | **Lower GI** | **Lung** | **Skin** | **Upper GI** | **Urological** | **All** |
| --- | --- | --- | --- | --- | --- | --- | --- | --- | --- | --- | --- |
| Excluded | n | 126 | 207 | 29 | 158 | 268 | 80 | 92 | 207 | 42 | 1209 |
| Included | n | 2199 | 2953 | 108 | 2091 | 2388 | 394 | 1167 | 1414 | 541 | 13255 |
| Excluded | Age, median [IQR] | 47.5 [35.0–60.8] | 56.0 [48.0–61.0] | 65.0 [57.2–72.0] | 57.0 [50.0–69.0] | 66.0 [55.0–74.0] | 71.0 [62.0–76.5] | 61.0 [48.0–74.0] | 63.0 [54.0–71.8] | 71.0 [63.0–76.0] | 60.0 [50.0–71.0] |
| Included | Age, median [IQR] | 48.0 [38.0–60.0] | 56.0 [51.0–64.0] | 66.0 [53.8–75.0] | 61.0 [48.0–72.0] | 68.0 [58.0–76.0] | 69.0 [59.0–76.0] | 67.0 [53.0–75.0] | 67.0 [56.0–75.0] | 68.0 [61.0–75.0] | 61.0 [50.0–72.0] |
| Excluded | Age: Unknown | 0 | 1 | 1 | 1 | 1 | 1 | 3 | 1 | 0 | 9 |
| Included | Age: Unknown | 0 | 0 | 0 | 0 | 0 | 0 | 0 | 0 | 0 | 0 |
| Excluded | Sex: F | 116 | 203 | 14 | 104 | 141 | 32 | 48 | 115 | 6 | 779 |
| Included | Sex: F | 2095 | 2952 | 49 | 1260 | 1283 | 179 | 614 | 862 | 74 | 9368 |
| Excluded | Sex: M | 7 | 2 | 15 | 53 | 127 | 48 | 40 | 90 | 36 | 418 |
| Included | Sex: M | 104 | 1 | 59 | 831 | 1105 | 215 | 553 | 552 | 467 | 3887 |
| Excluded | Sex: Unknown | 3 | 2 | 0 | 1 | 0 | 0 | 4 | 2 | 0 | 12 |
| Included | Sex: Unknown | 0 | 0 | 0 | 0 | 0 | 0 | 0 | 0 | 0 | 0 |
| Excluded | Ethnicity: Asian / Asian British | 3 | 7 | 1 | 10 | 9 | 3 | 0 | 11 | 0 | 44 |
| Included | Ethnicity: Asian / Asian British | 26 | 52 | 1 | 65 | 33 | 4 | 4 | 46 | 26 | 257 |
| Excluded | Ethnicity: Black / African / Caribbean / Black British | 0 | 0 | 0 | 5 | 0 | 0 | 0 | 2 | 0 | 7 |
| Included | Ethnicity: Black / African / Caribbean / Black British | 13 | 20 | 0 | 12 | 10 | 7 | 3 | 5 | 8 | 78 |
| Excluded | Ethnicity: Other ethnic group | 2 | 3 | 0 | 1 | 2 | 2 | 1 | 3 | 0 | 14 |
| Included | Ethnicity: Other ethnic group | 12 | 20 | 2 | 21 | 9 | 0 | 11 | 8 | 5 | 88 |
| Excluded | Ethnicity: White | 73 | 144 | 21 | 91 | 172 | 59 | 58 | 110 | 20 | 748 |
| Included | Ethnicity: White | 707 | 1039 | 53 | 1111 | 736 | 194 | 608 | 633 | 486 | 5567 |
| Excluded | Ethnicity: Unknown | 48 | 53 | 7 | 51 | 85 | 16 | 33 | 81 | 22 | 396 |
| Included | Ethnicity: Unknown | 1441 | 1822 | 52 | 882 | 1600 | 189 | 541 | 722 | 16 | 7265 |
| Excluded | Site: BTHFT | 32 | 69 | 8 | 19 | 16 | 1 | 15 | 32 | 0 | 192 |
| Included | Site: BTHFT | 0 | 36 | 5 | 43 | 0 | 5 | 0 | 18 | 0 | 107 |
| Excluded | Site: CHFT | 26 | 64 | 9 | 57 | 89 | 57 | 25 | 49 | 11 | 387 |
| Included | Site: CHFT | 169 | 695 | 29 | 741 | 455 | 155 | 331 | 442 | 63 | 3080 |
| Excluded | Site: HDFT | 2 | 6 | 1 | 5 | 3 | 1 | 2 | 5 | 0 | 25 |
| Included | Site: HDFT | 0 | 94 | 6 | 66 | 0 | 15 | 0 | 61 | 0 | 242 |
| Excluded | Site: LTHT | 25 | 18 | 4 | 24 | 78 | 6 | 15 | 46 | 11 | 227 |
| Included | Site: LTHT | 574 | 246 | 15 | 341 | 290 | 28 | 288 | 146 | 112 | 2040 |
| Excluded | Site: MYTT | 41 | 50 | 7 | 53 | 82 | 15 | 35 | 75 | 20 | 378 |
| Included | Site: MYTT | 1456 | 1882 | 53 | 900 | 1643 | 191 | 548 | 747 | 366 | 7786 |

To investigate the impact on performance of the patients excluded from the analysis, a sensitivity analysis was performed. This analysis was chosen to simulate the reasonable worst-case scenario, where the test is not informative for the excluded patients for whom data is not available. Where outcomes and PinPoint Test results were available for excluded patients, these were used. For excluded patients without an outcome, the patient was assigned to the cancer class with probability of the observed cancer prevalence for the relevant pathway. For excluded patients for whom a PinPoint Test result was not available, a value was chosen at random with replacement from the set of previously observed PinPoint Test results from the relevant pathway. The excluded patients were then added to the patients included in the analysis and the AUC for the combined set of patients was calculated. The process of assigning outcomes and PinPoint Test results and calculating the AUC was repeated 1000 times. Supplemental Figure 8 shows the difference between the median AUC for the combined patients and the AUC for only the included patients, for each pathway.


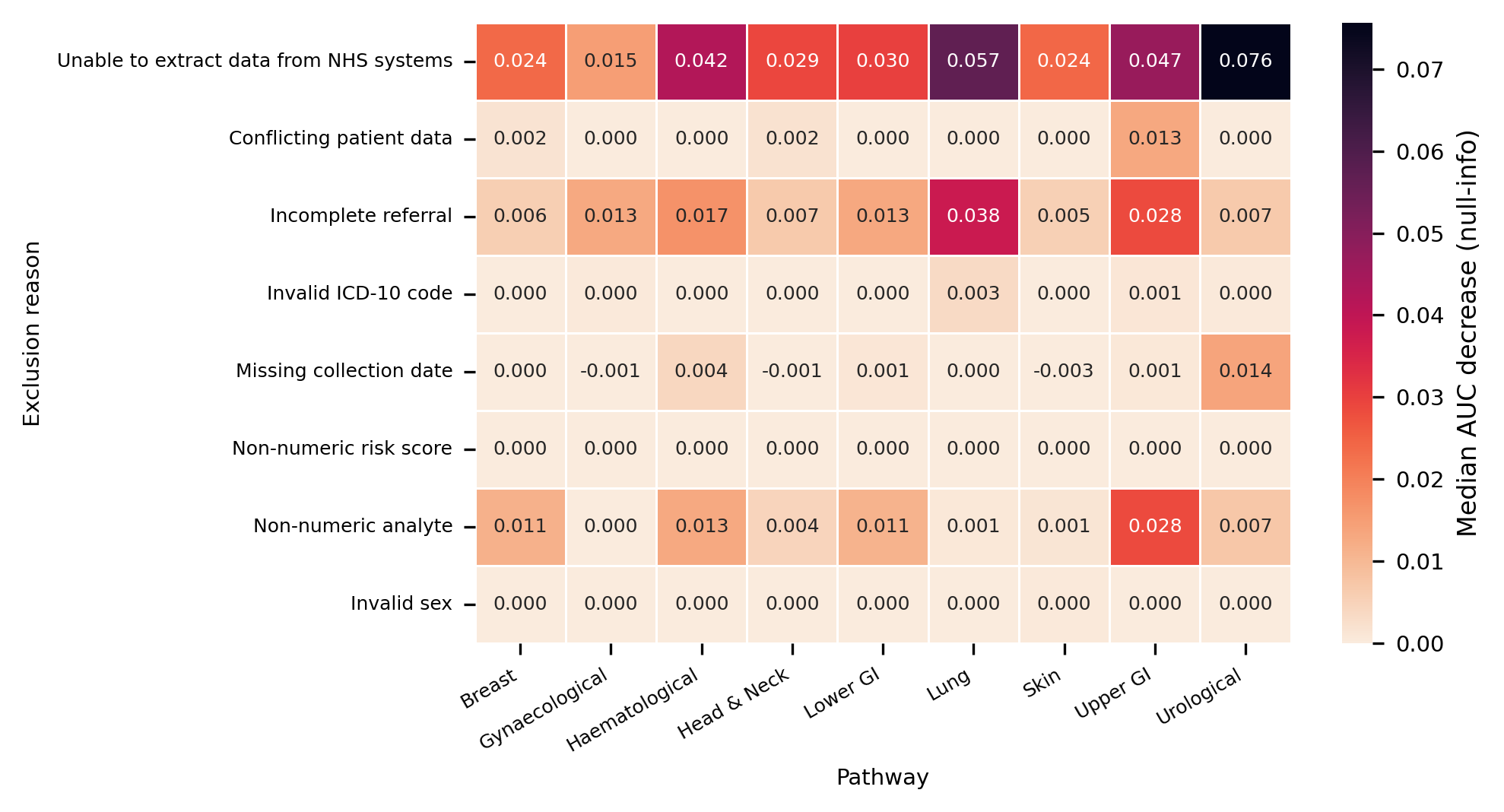


Supplemental Figure 8: Heatmap of the decrease in AUC between the included patients and the median AUC for a combined dataset of included and simulated excluded patients, for each pathway.

# Subgroup Analyses

Supplemental Tables 16, 17, and 18 give the results of the subgroup analyses of performance broken down by sex, ethnicity, and age respectively.

Supplemental Table 16: Performance of PinPoint Test v1.2 for each sex subgroup, for each USC pathway.

| **Pathway** | **Sex** | **Number of patients** | **Number of cancer patients** | **Number of non-cancer patients** | **AUC (95% CI) of PinPoint Test results** |
| --- | --- | --- | --- | --- | --- |
| Breast |  |  |  |  |  |
|  | Female | 2095 | 104 | 1991 | 0.73 (0.68–0.78) |
|  | Male | 104 | 3 | 101 | 0.65 (0.29–1.00) |
| Gynaecological |  |  |  |  |  |
|  | Female | 2952 | 114 | 2838 | 0.81 (0.77–0.85) |
|  | Male | 1 | 0 | 1 | Insufficient data |
| Haematological |  |  |  |  |  |
|  | Female | 49 | 9 | 40 | 0.66 (0.49–0.84) |
|  | Male | 59 | 12 | 47 | 0.67 (0.50–0.83) |
| Head and Neck |  |  |  |  |  |
|  | Female | 1260 | 34 | 1226 | 0.69 (0.60–0.79) |
|  | Male | 831 | 63 | 768 | 0.67 (0.60–0.74) |
| Lower GI |  |  |  |  |  |
|  | Female | 1283 | 39 | 1244 | 0.75 (0.68–0.83) |
|  | Male | 1105 | 54 | 1051 | 0.68 (0.60–0.76) |
| Lung |  |  |  |  |  |
|  | Female | 179 | 48 | 131 | 0.83 (0.76–0.90) |
|  | Male | 215 | 55 | 160 | 0.74 (0.67–0.82) |
| Skin |  |  |  |  |  |
|  | Female | 614 | 65 | 549 | 0.60 (0.52–0.67) |
|  | Male | 553 | 82 | 471 | 0.59 (0.53–0.65) |
| Upper GI |  |  |  |  |  |
|  | Female | 862 | 30 | 832 | 0.82 (0.72–0.91) |
|  | Male | 552 | 49 | 503 | 0.86 (0.81–0.91) |
| Urological |  |  |  |  |  |
|  | Female | 74 | 6 | 68 | 0.69 (0.41–0.98) |
|  | Male | 467 | 104 | 363 | 0.72 (0.66–0.78) |

Supplemental Table 17: Performance of PinPoint Test v1.2 for each ethnicity subgroup, for each USC pathway. Patients without ethnicity data were excluded from this analysis.

| **Pathway** | **Ethnicity** | **Number of patients** | **Number of cancer patients** | **Number of non-cancer patients** | **AUC (95% CI) of PinPoint Test results** |
| --- | --- | --- | --- | --- | --- |
| Breast |  |  |  |  |  |
|  | White | 707 | 37 | 670 | 0.71 (0.62–0.80) |
|  | Asian / Asian British | 26 | 1 | 25 | Insufficient data |
|  | Black / African / Caribbean / Black British | 13 | 0 | 13 | Insufficient data |
|  | Other ethnic group | 12 | 0 | 12 | Insufficient data |
| Gynaecological |  |  |  |  |  |
|  | White | 1039 | 41 | 998 | 0.87 (0.82–0.92) |
|  | Asian / Asian British | 52 | 1 | 51 | Insufficient data |
|  | Black / African / Caribbean / Black British | 20 | 1 | 19 | Insufficient data |
|  | Other ethnic group | 20 | 1 | 19 | Insufficient data |
| Haematological |  |  |  |  |  |
|  | White | 53 | 12 | 41 | 0.54 (0.35–0.73) |
|  | Asian / Asian British | 1 | 1 | 0 | Insufficient data |
|  | Black / African / Caribbean / Black British | 0 | 0 | 0 | Insufficient data |
|  | Other ethnic group | 2 | 0 | 2 | Insufficient data |
| Head and Neck |  |  |  |  |  |
|  | White | 1111 | 62 | 1049 | 0.72 (0.66–0.78) |
|  | Asian / Asian British | 65 | 3 | 62 | 0.39 (0.00–0.88) |
|  | Black / African / Caribbean / Black British | 12 | 2 | 10 | 0.90 (0.66–1.00) |
|  | Other ethnic group | 21 | 0 | 21 | Insufficient data |
| Lower GI |  |  |  |  |  |
|  | White | 736 | 27 | 709 | 0.68 (0.58–0.78) |
|  | Asian / Asian British | 33 | 1 | 32 | Insufficient data |
|  | Black / African / Caribbean / Black British | 10 | 0 | 10 | Insufficient data |
|  | Other ethnic group | 9 | 0 | 9 | Insufficient data |
| Lung |  |  |  |  |  |
|  | White | 194 | 61 | 133 | 0.76 (0.69–0.83) |
|  | Asian / Asian British | 4 | 1 | 3 | Insufficient data |
|  | Black / African / Caribbean / Black British | 7 | 1 | 6 | Insufficient data |
|  | Other ethnic group | 0 | 0 | 0 | Insufficient data |
| Skin |  |  |  |  |  |
|  | White | 608 | 89 | 519 | 0.60 (0.53–0.66) |
|  | Asian / Asian British | 4 | 0 | 4 | Insufficient data |
|  | Black / African / Caribbean / Black British | 3 | 0 | 3 | Insufficient data |
|  | Other ethnic group | 11 | 0 | 11 | Insufficient data |
| Upper GI |  |  |  |  |  |
|  | White | 633 | 32 | 601 | 0.84 (0.75–0.93) |
|  | Asian / Asian British | 46 | 2 | 44 | 0.97 (0.91–1.00) |
|  | Black / African / Caribbean / Black British | 8 | 0 | 8 | Insufficient data |
|  | Other ethnic group | 5 | 0 | 5 | Insufficient data |
| Urological |  |  |  |  |  |
|  | White | 486 | 106 | 380 | 0.73 (0.67–0.78) |
|  | Asian / Asian British | 26 | 0 | 26 | Insufficient data |
|  | Black / African / Caribbean / Black British | 8 | 2 | 6 | Insufficient data |
|  | Other ethnic group | 5 | 0 | 5 | Insufficient data |

Supplemental Table 18: Performance of PinPoint Test v1.2 for each age subgroup, for each USC pathway.

| **Pathway** | **Age quartile** | **Number of patients** | **Number of cancer patients** | **Number of non-cancer patients** | **AUC (95% CI) of PinPoint Test results** |
| --- | --- | --- | --- | --- | --- |
| Breast |  |  |  |  |  |
|  | Q1 (18 ≤ age < 38) | 587 | 7 | 580 | 0.57 (0.31–0.83) |
|  | Q2 (38 ≤ age < 48) | 537 | 21 | 516 | 0.59 (0.45–0.73) |
|  | Q3 (48 ≤ age < 60) | 549 | 24 | 525 | 0.60 (0.46–0.73) |
|  | Q4 (60 ≤ age ≤ 92) | 526 | 55 | 471 | 0.66 (0.59–0.74) |
| Gynaecological |  |  |  |  |  |
|  | Q1 (19 ≤ age < 51) | 805 | 11 | 794 | 0.76 (0.60–0.93) |
|  | Q2 (51 ≤ age < 56) | 705 | 10 | 695 | 0.85 (0.70–1.00) |
|  | Q3 (56 ≤ age < 64) | 749 | 30 | 719 | 0.78 (0.68–0.87) |
|  | Q4 (64 ≤ age ≤ 94) | 694 | 63 | 631 | 0.67 (0.60–0.75) |
| Haematological |  |  |  |  |  |
|  | Q1 (19 ≤ age < 53) | 27 | 1 | 26 | Insufficient data |
|  | Q2 (53 ≤ age < 66) | 32 | 7 | 25 | 0.60 (0.31–0.89) |
|  | Q3 (66 ≤ age < 75) | 23 | 6 | 17 | 0.48 (0.21–0.75) |
|  | Q4 (75 ≤ age ≤ 89) | 26 | 7 | 19 | 0.46 (0.16–0.75) |
| Head and Neck |  |  |  |  |  |
|  | Q1 (18 ≤ age < 48) | 534 | 9 | 525 | 0.71 (0.49–0.93) |
|  | Q2 (48 ≤ age < 61) | 540 | 25 | 515 | 0.69 (0.59–0.80) |
|  | Q3 (61 ≤ age < 72) | 510 | 25 | 485 | 0.68 (0.56–0.79) |
|  | Q4 (72 ≤ age ≤ 96) | 507 | 38 | 469 | 0.69 (0.61–0.78) |
| Lower GI |  |  |  |  |  |
|  | Q1 (18 ≤ age < 58) | 631 | 19 | 612 | 0.73 (0.61–0.86) |
|  | Q2 (58 ≤ age < 68) | 601 | 25 | 576 | 0.72 (0.63–0.81) |
|  | Q3 (68 ≤ age < 76) | 619 | 24 | 595 | 0.71 (0.61–0.81) |
|  | Q4 (76 ≤ age ≤ 101) | 537 | 25 | 512 | 0.76 (0.67–0.85) |
| Lung |  |  |  |  |  |
|  | Q1 (23 ≤ age < 59) | 101 | 12 | 89 | 0.81 (0.66–0.97) |
|  | Q2 (59 ≤ age < 69) | 99 | 24 | 75 | 0.69 (0.56–0.83) |
|  | Q3 (69 ≤ age < 76) | 101 | 35 | 66 | 0.81 (0.72–0.90) |
|  | Q4 (76 ≤ age ≤ 93) | 93 | 32 | 61 | 0.71 (0.60–0.83) |
| Skin |  |  |  |  |  |
|  | Q1 (18 ≤ age < 53) | 305 | 21 | 284 | 0.57 (0.45–0.69) |
|  | Q2 (53 ≤ age < 67) | 298 | 35 | 263 | 0.58 (0.48–0.68) |
|  | Q3 (67 ≤ age < 75) | 282 | 39 | 243 | 0.57 (0.47–0.67) |
|  | Q4 (75 ≤ age ≤ 98) | 282 | 52 | 230 | 0.49 (0.40–0.57) |
| Upper GI |  |  |  |  |  |
|  | Q1 (21 ≤ age < 56) | 357 | 7 | 350 | 0.97 (0.93–1.00) |
|  | Q2 (56 ≤ age < 67) | 391 | 15 | 376 | 0.68 (0.52–0.84) |
|  | Q3 (67 ≤ age < 75) | 335 | 28 | 307 | 0.86 (0.78–0.94) |
|  | Q4 (75 ≤ age ≤ 95) | 331 | 29 | 302 | 0.87 (0.80–0.94) |
| Urological |  |  |  |  |  |
|  | Q1 (26 ≤ age < 61) | 144 | 14 | 130 | 0.71 (0.55–0.87) |
|  | Q2 (61 ≤ age < 68) | 141 | 30 | 111 | 0.67 (0.56–0.78) |
|  | Q3 (68 ≤ age < 75) | 138 | 42 | 96 | 0.75 (0.67–0.84) |
|  | Q4 (75 ≤ age ≤ 90) | 118 | 24 | 94 | 0.65 (0.51–0.79) |

# Checklists

STROBE^14^ and STARD^15^ checklists were used for this paper. The completed checklists are provided as separate supplemental files.
