# Supplementary material for: Real-World Validation of PinPoint Blood Tests in the NHS: Multivariable Machine Learning to Predict Cancer Risk in Primary Care Urgent Referrals
Source: Mayo Clin Proc Digit Health. 2026 Jun 10;4(3):100382. doi: 10.1016/j.mcpdig.2026.100382 (PMC13325911; doi:10.1016/j.mcpdig.2026.100382)
Supplement: STARD Checklist [file mmc2.docx]

The STARD reporting checklist

For checking that diagnostic accuracy study articles can be understood and used by everyone

| 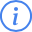 Note |
| --- |
| If you have not used a reporting guideline before, read about [how and why to use them](https:/resources.equator-network.org/about/reporting-guidelines.html) and check whether STARD is the [most applicable reporting guideline](https:/resources.equator-network.org/reporting-guidelines/stard/index.html?#applicability) for your work.  Reporting guidelines are most useful when used early in research. When writing a manuscript or application, consider using the [Full Guidance](https:/resources.equator-network.org/reporting-guidelines/stard/index.html) where you’ll see explanations and examples for each item.  After writing, demonstrate adherence by completing this checklist:   1. Specify where each item is described (see [Note 1](#sec-specify)). 2. Cite this checklist (See [Note 2](#sec-cite)). 3. Include your completed checklist as a supplement when submitting to a journal so that future readers can use it to find information. |

|  | Item Description | Location (or reason for not reporting) |
| --- | --- | --- |
| **Title or abstract** |  |  |
| [1. Identification as a study of diagnostic accuracy](https:/resources.equator-network.org/reporting-guidelines/stard/items/identification.html?utm_source=stard&utm_medium=checklist&utm_campaign=STARD_2015_v1_1) | Identification as a study of diagnostic accuracy using at least one measure of accuracy (such as sensitivity, specificity, predictive values, or AUC). | Abstract |
| **Abstract** |  |  |
| [2. Abstract](https:/resources.equator-network.org/reporting-guidelines/stard/items/abstract.html?utm_source=stard&utm_medium=checklist&utm_campaign=STARD_2015_v1_1) | Structured summary of study design, methods, results and conclusions (for specific guidance, see STARD for Abstracts). | Abstract |
| **Introduction** |  |  |
| [3. Background](https:/resources.equator-network.org/reporting-guidelines/stard/items/background.html?utm_source=stard&utm_medium=checklist&utm_campaign=STARD_2015_v1_1) | Scientific and clinical background, including the intended use and clinical role of the index test. | Introduction, paragraphs 2-4 |
| [4. Objectives](https:/resources.equator-network.org/reporting-guidelines/stard/items/objectives.html?utm_source=stard&utm_medium=checklist&utm_campaign=STARD_2015_v1_1) | Study objectives and hypotheses. | Introduction, paragraph 4 |
| **Methods** |  |  |
| [5. Study design](https:/resources.equator-network.org/reporting-guidelines/stard/items/study-design.html?utm_source=stard&utm_medium=checklist&utm_campaign=STARD_2015_v1_1) | Whether data collection was planned before the index test and reference standard were performed (prospective study) or after (retrospective study). | Methods, Methodological Design and Source of Data, paragraph 1 |
| Participants |  |  |
| [6. Eligibility criteria](https:/resources.equator-network.org/reporting-guidelines/stard/items/eligibility-criteria.html?utm_source=stard&utm_medium=checklist&utm_campaign=STARD_2015_v1_1) | Eligibility criteria. | Methods, Participants |
| [7. Identifying eligible participants](https:/resources.equator-network.org/reporting-guidelines/stard/items/identifying-eligible-participants.html?utm_source=stard&utm_medium=checklist&utm_campaign=STARD_2015_v1_1) | On what basis potentially eligible participants were identified (such as symptoms, results from previous tests, inclusion in registry). | Methods, Methodological Design and Source of Data, paragraph 1  Methods, Participants |
| [8. Setting, location, and dates](https:/resources.equator-network.org/reporting-guidelines/stard/items/setting-location-dates.html?utm_source=stard&utm_medium=checklist&utm_campaign=STARD_2015_v1_1) | Where and when potentially eligible participants were identified (setting, location and dates). | Methods, Methodological Design and Source of Data, paragraphs 1 and 3  Supplementary Materials, Enrolment End Dates |
| [9. Consecutive, random or convenience series](https:/resources.equator-network.org/reporting-guidelines/stard/items/consecutive-random-convenience-series.html?utm_source=stard&utm_medium=checklist&utm_campaign=STARD_2015_v1_1) | Whether participants formed a consecutive, random or convenience series. | Methods, Methodological Design and Source of Data, paragraph 1 |
| Test Methods |  |  |
| [10. Index test & Reference standard](https:/resources.equator-network.org/reporting-guidelines/stard/items/index-test.html?utm_source=stard&utm_medium=checklist&utm_campaign=STARD_2015_v1_1) | 10a. Index test  10b. Reference standard | 10a: Methods, Methodological Design and Source of Data, paragraph 4  Methods, Statistical Analysis Methods, Analysis Methods, paragraph 2  10b: Not applicable as the reference standard is standard care |
| [11. Reference standard rationale](https:/resources.equator-network.org/reporting-guidelines/stard/items/reference-standard-rationale.html?utm_source=stard&utm_medium=checklist&utm_campaign=STARD_2015_v1_1) | Rationale for choosing the reference standard (if alternatives exist). | NA, no alternatives available |
| [12. Index test and reference standard cut-offs or categories](https:/resources.equator-network.org/reporting-guidelines/stard/items/index-test-cut-offs-categories.html?utm_source=stard&utm_medium=checklist&utm_campaign=STARD_2015_v1_1) | 12a. Definition of and rationale for test positivity cut-offs or result categories of the index test, distinguishing prespecified from exploratory.  12b. Definition of and rationale for test positivity cut-offs or result categories of the reference standard, distinguishing prespecified from exploratory. | 12a: Methods, Statistical Analysis Methods, Model Development, paragraph 1  Methods, Statistical Analysis Methods, Analysis Methods, paragraph 7  12b: Not applicable as the reference standard is standard care |
| [13. Information available to performers or readers of the index test and reference standard assessors](https:/resources.equator-network.org/reporting-guidelines/stard/items/information-available-index.html?utm_source=stard&utm_medium=checklist&utm_campaign=STARD_2015_v1_1) | 13a. Whether clinical information and reference standard results were available to the performers or readers of the index test.  13b. Whether clinical information and index test results were available to the assessors of the reference standard. | 13a: Methods, Outcome  13b: Methods, Methodological Design and Source of Data, paragraph 5 |
| Analysis |  |  |
| [14. Analysis methods](https:/resources.equator-network.org/reporting-guidelines/stard/items/analysis-methods.html?utm_source=stard&utm_medium=checklist&utm_campaign=STARD_2015_v1_1) | Methods for estimating or comparing measures of diagnostic accuracy. | Methods, Statistical Analysis Methods, Analysis Methods  Supplementary Materials, Calibration  Supplementary Materials, Temporal Analysis  Supplementary Materials, Exclusions from Analysis  Supplementary Analysis, Subgroup Analyses |
| [15. Indeterminate results](https:/resources.equator-network.org/reporting-guidelines/stard/items/indeterminate-results.html?utm_source=stard&utm_medium=checklist&utm_campaign=STARD_2015_v1_1) | How indeterminate index test or reference standard results were handled. | NA, indeterminate results were not observed |
| [16. Missing data](https:/resources.equator-network.org/reporting-guidelines/stard/items/missing-data.html?utm_source=stard&utm_medium=checklist&utm_campaign=STARD_2015_v1_1) | How missing data on the index test and reference standard were handled. | Methods, Management of Missing Data |
| [17. Variability](https:/resources.equator-network.org/reporting-guidelines/stard/items/variability.html?utm_source=stard&utm_medium=checklist&utm_campaign=STARD_2015_v1_1) | Any analyses of variability in diagnostic accuracy, distinguishing prespecified from exploratory. | Methods, Statistical Analysis Methods, Analysis Methods, paragraphs 4-5  Supplementary Materials, Temporal Analysis |
| [18. Intended sample size](https:/resources.equator-network.org/reporting-guidelines/stard/items/intended-sample-size.html?utm_source=stard&utm_medium=checklist&utm_campaign=STARD_2015_v1_1) | Intended sample size and how it was determined. | Methods, Sample Size  Supplementary Materials, Sample Size Calculations |
| **Results** |  |  |
| Participants |  |  |
| [19. Participant flow diagram](https:/resources.equator-network.org/reporting-guidelines/stard/items/flow-diagram.html?utm_source=stard&utm_medium=checklist&utm_campaign=STARD_2015_v1_1) | Flow of participants, using a diagram. | Results, Participants and Demographics, paragraph 1  Figure 1 |
| [20. Baseline characteristics](https:/resources.equator-network.org/reporting-guidelines/stard/items/baseline-characteristics.html?utm_source=stard&utm_medium=checklist&utm_campaign=STARD_2015_v1_1) | Baseline demographic and clinical characteristics of participants. | Results, Participants and Demographics, paragraph 2  Table 1 |
| [21a. Participants with and without the target condition](https:/resources.equator-network.org/reporting-guidelines/stard/items/participants-with-target-condition.html?utm_source=stard&utm_medium=checklist&utm_campaign=STARD_2015_v1_1) | 21a. Distribution of severity of disease in those with the target condition.  21b. Distribution of alternative diagnoses in those without the target condition | 21a: Data not available  21b: Supplementary Materials, Outcomes |
| [22. Time interval](https:/resources.equator-network.org/reporting-guidelines/stard/items/time-interval.html?utm_source=stard&utm_medium=checklist&utm_campaign=STARD_2015_v1_1) | Time interval and any clinical interventions between index test and reference standard. | Data not available |
| Test Results |  |  |
| [23. Index test and reference standard results](https:/resources.equator-network.org/reporting-guidelines/stard/items/index-reference-results.html?utm_source=stard&utm_medium=checklist&utm_campaign=STARD_2015_v1_1) | Cross tabulation of the index test results (or their distribution) by the results of the reference standard. | Supplementary Materials, Confusion Matrices |
| [24. Estimates of accuracy](https:/resources.equator-network.org/reporting-guidelines/stard/items/estimates-of-accuracy.html?utm_source=stard&utm_medium=checklist&utm_campaign=STARD_2015_v1_1) | Estimates of diagnostic accuracy and their precision (such as 95% CIs). | Results, Performance by Pathway, paragraphs 1 and 2 |
| [25. Adverse events](https:/resources.equator-network.org/reporting-guidelines/stard/items/adverse-events.html?utm_source=stard&utm_medium=checklist&utm_campaign=STARD_2015_v1_1) | Any adverse events from performing the index test or the reference standard. | Results, Adverse Events |
| **Discussion** |  |  |
| [26. Limitations](https:/resources.equator-network.org/reporting-guidelines/stard/items/limitations.html?utm_source=stard&utm_medium=checklist&utm_campaign=STARD_2015_v1_1) | Study limitations, including sources of potential bias, statistical uncertainty and generalisability. | Discussion, Limitations of the Work |
| [27. Implications for Practice](https:/resources.equator-network.org/reporting-guidelines/stard/items/implications-for-practice.html?utm_source=stard&utm_medium=checklist&utm_campaign=STARD_2015_v1_1) | Implications for practice, including the intended use and clinical role of the index test. | Discussion, Implications for Policy and Practice |
| **Other information** |  |  |
| [28. Registration](https:/resources.equator-network.org/reporting-guidelines/stard/items/registration.html?utm_source=stard&utm_medium=checklist&utm_campaign=STARD_2015_v1_1) | Registration number and name of registry. | Not available. Registered as NHS service evaluation |
| [29. Protocol](https:/resources.equator-network.org/reporting-guidelines/stard/items/protocol.html?utm_source=stard&utm_medium=checklist&utm_campaign=STARD_2015_v1_1) | Where the full study protocol can be accessed. | Protocol |
| [30. Funding](https:/resources.equator-network.org/reporting-guidelines/stard/items/funding.html?utm_source=stard&utm_medium=checklist&utm_campaign=STARD_2015_v1_1) | Sources of funding and other support; role of funders. | Declaration of Interests, Role of the Funding Source  Acknowledgements |

## 1 How to specify where content is

Tell the reader where they can find information. E.g.,

- Results; paragraph 2
- Methods, Participants; paragraphs 1 & 2.
- Table 3
- Supplement B, para. 4

If you have chosen not to describe an item, explain why. You can do this in the checklist, or as a note below it.

You can describe items in the article body, or in tables, figures, or supplementary materials, and should prioritize items you feel are most important to your intended audience. The order of items in your manuscript does not need to match the order of items in this checklist. You can decide how best to structure your work.

## 2 How to cite

Describe how you used STARD at the end of your Methods section, referencing the resources you used e.g.,

‘We used the STARD reporting guideline(1) to draft this manuscript, and the STARD reporting checklist(2) when editing, included in supplement A’

If you use a reporting checklist, remember to include it as a supplement when publishing so that readers can easily find information and see how you have interpreted the guidance.

1. Bossuyt PM, Reitsma JB, Bruns DE, Gatsonis CA, Glasziou PP, Irwig L, et al. STARD 2015: An updated list of essential items for reporting diagnostic accuracy studies. BMJ [Internet]. 2015 Oct;351:h5527. Available from: <https://www.bmj.com/content/351/bmj.h5527>

2. Bossuyt PM, Reitsma JB, Bruns DE, Gatsonis CA, Glasziou PP, Irwig L, et al. The STARD reporting checklist. In: Harwood J, Albury C, Beyer J de, Schlüssel M, Collins G, editors. The EQUATOR network reporting guideline platform [Internet]. The UK EQUATOR Centre; 2025. Available from: [https:/resources.equator-network.org/reporting-guidelines/stard/stard-checklist.docx](https://https:/resources.equator-network.org/reporting-guidelines/stard/stard-checklist.docx)
